# Unraveling the RKIP–YY1 axis: immune crosstalk in the pathogenesis of metabolic disorders

**DOI:** 10.3389/fimmu.2025.1675699

**Published:** 2025-12-11

**Authors:** Fawaz Alzaid, Hossein Arefanian, Fatemah Bahman, Shaima Albeloushi, Ghadeer Alhamar, Anwar Mohammad, Amal Hasan, Ashraf Al Madhoun, Rasheed Ahmad, Fahd Al-Mulla

**Affiliations:** 1 Dasman Diabetes Institute, Kuwait City, Kuwait; 2 INSERM UMR-S1151, CNRS UMR-S8253, Université Paris Cité, Institut Necker Enfants Malades, Paris, France

**Keywords:** RKIP, YY1, immunity, metabolic disease, inflammation

## Abstract

Metabolic diseases, including obesity, type 2 diabetes, and cardiovascular disorders, are increasingly recognized as chronic inflammatory conditions driven by dysregulated immune-metabolic interactions. Two pivotal regulators of this crosstalk are Raf kinase inhibitor protein (RKIP) and the transcription factor Yin Yang 1 (YY1), which coordinate inflammatory signaling and metabolic stress responses across multiple tissues. RKIP exerts protective, anti-inflammatory effects by antagonizing the MAPK and NF-κB pathways, thereby preserving tissue homeostasis under metabolic stress. In contrast, YY1 acts as a context-dependent transcriptional regulator that promotes inflammatory gene programs, contributes to maladaptive immune cell differentiation, and exacerbates metabolic dysfunction. Notably, RKIP and YY1 are reciprocally regulated: RKIP suppresses YY1 expression via NF-κB inhibition, whereas YY1 represses RKIP transcription through a Snail-dependent feedback loop. In metabolic disease states, this balance is disrupted, RKIP is downregulated, and YY1 is upregulated, leading to heightened immune activation, cytokine production, and tissue damage. Therefore, we propose that RKIP and YY1 represent two opposing yet dynamically coordinated regulators of immunometabolic balance, functioning as a molecular rheostat that determines whether immune responses shift toward inflammation or resolution under metabolic stress. This review synthesizes current insights into the molecular structures, signaling pathways, and tissue-specific functions of RKIP and YY1, emphasizing their interplay in shaping immune responses in metabolic disorders. We further discuss emerging therapeutic approaches aimed at restoring RKIP–YY1 homeostasis to mitigate chronic inflammation and metabolic pathology.

## Graphical Abstract

**Figure:**
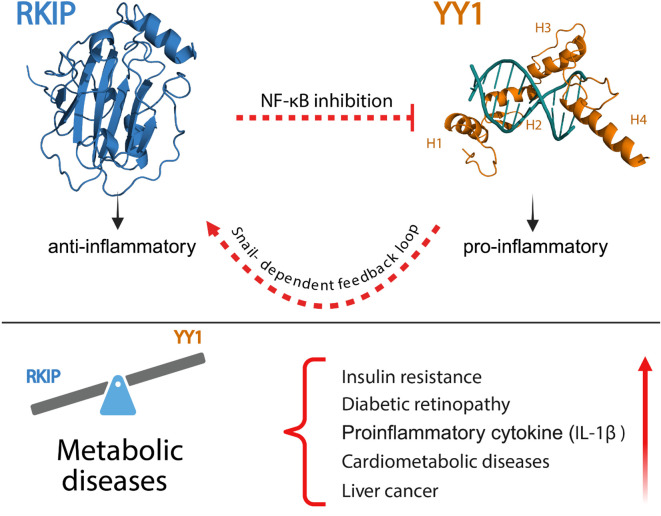

## 1 Introduction

### 1.1 Overview of metabolic diseases and underlying mechanisms

Metabolic diseases comprise a group of disorders marked by disruptions in the body’s normal metabolic functions. These functions include the conversion of food into energy and the regulation of cellular and biochemical pathways critical for maintaining both tissue-specific and systemic homeostasis. Dysregulation of these pathways, driven by genetic, environmental, or lifestyle factors, can lead to the onset of metabolic diseases. Among the most common are obesity, diabetes mellitus, and cardiovascular diseases (1).

Obesity is a chronic condition marked by excessive accumulation of adipose tissue, resulting from an imbalance between energy intake and expenditure (2). It is strongly associated with systemic inflammation, insulin resistance, and an increased risk of comorbidities such as type 2 diabetes (T2D) and hypertension (3). Diabetes mellitus refers to a group of metabolic disorders characterized by chronic hyperglycemia due to defects in insulin secretion and/or function. It is primarily classified into type 1 diabetes (T1D), caused by autoimmune destruction of pancreatic β-cells, and T2D, which involves insulin resistance and relative insulin deficiency (4). Cardiovascular diseases (CVDs) encompass a range of disorders affecting the heart and blood vessels, including coronary artery disease, stroke, and hypertension. These conditions are closely associated with metabolic abnormalities such as dyslipidemia, hyperglycemia, and obesity, and are often components of the broader metabolic syndrome (5). A comprehensive understanding of the underlying mechanisms driving metabolic diseases is essential for advancing research, as well as improving prevention, diagnosis, and treatment strategies. These disorders are multifactorial in nature, arising from complex interactions between genetic predisposition, environmental influences, and behavioral factors. Central to the pathophysiology of most metabolic conditions is the dysregulation of key pathways involving insulin signaling, adipokine secretion, inflammation, oxidative stress, and mitochondrial function (6). Elucidating these mechanisms enables the identification of early biomarkers and the development of therapeutic targets. For example, a deeper understanding of hyperglycemia, insulin resistance, and chronic low-grade inflammation in T2D has led to the development of therapeutic agents such as metformin and GLP-1 receptor agonists (7, 8). Similarly, advances in the understanding of lipid metabolism and endothelial dysfunction have shaped modern cardiovascular pharmacotherapy (9).

Moreover, mechanistic insights support personalized medicine by enabling patient stratification based on molecular profiles, thereby enhancing treatment efficacy and minimizing adverse effects. Coordinated efforts that integrate mechanistic research with epidemiological studies can inform public health strategies aimed at addressing modifiable risk factors, including diet, physical activity, and socioeconomic determinants. Ultimately, a deeper understanding of disease mechanisms bridges the gap between basic science and clinical practice, accelerating the translation of discoveries into improved health outcomes (10).

### 1.2 Immune crosstalk in metabolic diseases

Immune crosstalk refers to the dynamic, bidirectional interaction between immune cells and metabolic tissues that shapes both immune responses and metabolic homeostasis. In the context of metabolic disease, this crosstalk becomes dysregulated, contributing significantly to disease progression (11). Key metabolic organs, including adipose tissue, liver, skeletal muscle, and the gut, serve as important sites of immune surveillance. Under conditions of metabolic stress, such as overnutrition or lipid overload, immune cells infiltrate these tissues and shift toward pro-inflammatory phenotypes (12–14). This response leads to chronic low-grade inflammation, a hallmark of many metabolic disorders, which perpetuates insulin resistance, endothelial dysfunction, and lipid abnormalities (15).

The immune system plays a dual role in metabolism, acting both as a regulator of homeostasis and a driver of inflammation. Macrophages, T cells, dendritic cells (DCs), and innate lymphoid cells are among the primary immune cell populations residing in metabolic tissues16. In lean individuals, these cells maintain a quiescent, tolerogenic profile that supports insulin sensitivity and metabolic balance (16–18). However, in obesity and during metabolic stress, the immune landscape shifts toward a pro-inflammatory state. This includes increased presence of M1-like macrophages and Th1 T cells, which secrete cytokines such as TNF, IL-6, and IFN-γ (19). These inflammatory mediators disrupt insulin signaling and contribute to tissue remodeling, fibrosis, and overall metabolic dysfunction (20). Understanding the immune-metabolic interface is critical for developing targeted therapies that can modulate immune responses to restore metabolic homeostasis without compromising host defense mechanisms.

### 1.3 Mediators of chronic inflammation in metabolic disease

Immune crosstalk plays a central role in the development of insulin resistance and broader metabolic dysfunction in conditions such as obesity and T2D (21). In metabolic tissues, including adipose tissue, liver, and skeletal muscle, chronic overnutrition induces cellular stress, lipid accumulation, and hypoxia. These stressors collectively trigger immune cell infiltration and activation (13, 22), leading to an imbalance between pro-inflammatory and anti-inflammatory signals (11).

**Figure 1:**
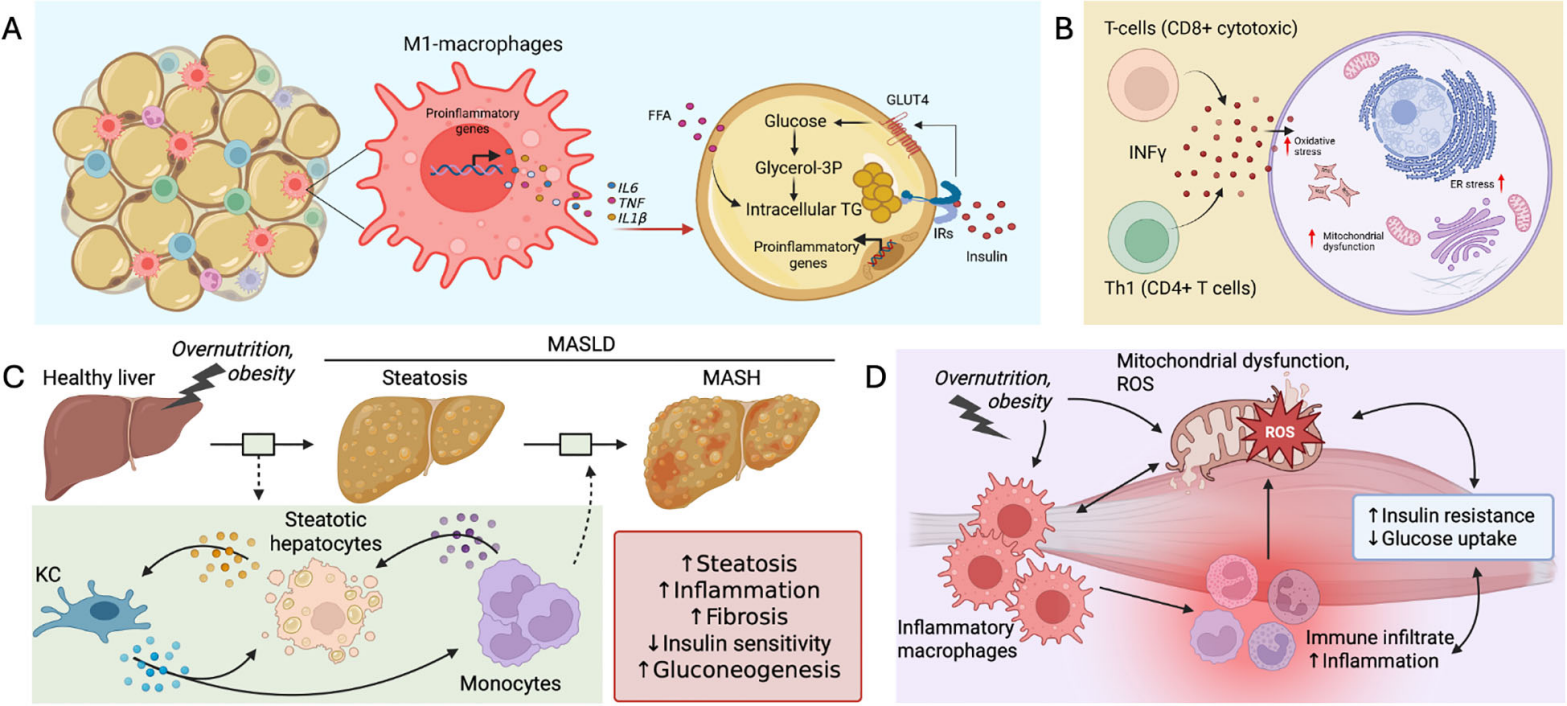
Obesity-driven immune–metabolic crosstalk and its contribution to insulin resistance and liver disease. Obesity promotes infiltration and activation of pro-inflammatory immune cells in key metabolic tissues, contributing to systemic insulin resistance and organ-specific pathology. **(A)** In adipose tissue, M1-polarized macrophages secrete inflammatory cytokines such as IL-6, TNF, and IL-1β in response to excess free fatty acids (FFAs), impairing insulin signaling and promoting lipid accumulation. **(B)** The T cell compartment (CD4^+^ Th1 and CD8^+^ T cells) also contributes by secreting IFN- γ, contributing to cellular stress, mitochondrial dysfunction. **(C)** In the liver, ectopic lipid storage in hepatocytes signals stress to Kupffer cells (KC). KCs signal to recruit monocytes that join myeloid cells in the tissue to propagate inflammation. This further impairs insulin sensitivity, increases gluconeogenesis, and aggravates mitochondrial dysfunction, promoting reactive oxygen species (ROS) production. A cycle that of inflammation and metabolic dysfunction leads to progression through the metabolic dysfunction-associated steatotic liver disease (MASLD) spectrum, to metabolic dysfunction-associated steatohepatitis. **(D)** In skeletal muscle, overnutrition and obesity leads to mitochondrial dysfunction, these signals are sensed by macrophages that undergo inflammatory polarization. Macrophages signal to recruit other immune cells that contribute to tissue inflammation, worsening insulin resistance and decrease glucose uptake capacity.

One of the earliest immune events is the accumulation of classically activated (M1-like) macrophages in adipose tissue, replacing the alternatively activated (M2-like) macrophages that are predominant under lean conditions. M1 macrophages secrete pro-inflammatory cytokines, including TNF, IL-6, and IL-1β, which interfere with insulin signaling by promoting serine phosphorylation of insulin receptor substrate (IRS) proteins, thereby impairing insulin action (18) (**Figure 1A**). In parallel, pro-inflammatory T cell subsets, particularly CD8+ cytotoxic T cells and CD4+ Th1 cells, accumulate and secrete IFN-γ, further amplifying the inflammatory environment (**Figure 1B**). This cytokine-driven inflammation exacerbates mitochondrial dysfunction, oxidative stress, and endoplasmic reticulum stress in metabolic cells, compounding insulin resistance (14, 23).

In the liver, Kupffer cells and recruited monocytes produce similar inflammatory mediators that stimulate gluconeogenesis, impair hepatic insulin sensitivity, and promote progression from simple steatosis to steatohepatitis (24) (**Figure 1C**). In skeletal muscle, inflammation driven by immune cell infiltration disrupts glucose uptake and mitochondrial function (25) (**Figure 1D**). Thus, chronic, unresolved inflammation driven by immune-metabolic crosstalk is a key contributor to the development and persistence of metabolic dysfunction.

Recent advances in immunometabolism have revealed that immune cell activation and metabolic reprogramming are tightly interconnected processes governed by shared signaling networks. Given the critical role of these inflammatory and metabolic pathways in maintaining cellular function, key regulator mechanisms have become the focus of intense investigation. Notably, insights from cancer biology, where both immune and metabolic networks are commonly dysregulated, have identified two key regulators: Raf kinase inhibitor protein (RKIP) and its counter-regulator Yin Yang 1 (YY1). These molecules have been extensively studied for their ability to modulate immune and metabolic function at the cellular level, either by interacting with multiple intracellular signaling pathways or by regulating transcriptional programs. RKIP predominantly acts as a suppressor of pro-inflammatory kinase cascades such as MAPK and NF-κB, whereas YY1 functions as a transcriptional amplifier that promotes metabolic gene expression and inflammatory mediator production. We propose that RKIP and YY1 act as antagonistic yet interdependent regulators of immunometabolic homeostasis, functioning as a molecular rheostat that determines the direction of immune responses toward inflammation or resolution under metabolic stress. In the sections that follow, we critically examine the emerging roles of RKIP and YY1 in the context of metabolic disease, highlight current knowledge gaps, and discuss potential therapeutic implications for obesity, T2D, and cardiovascular disorders.

#### 1.3.1 Raf kinase inhibitor protein structure-function dynamics

Raf Kinase Inhibitory Protein (RKIP), also known as phosphatidylcholine-binding protein 1 (PEBP1), belongs to the PEBP family, a group of evolutionarily conserved and ubiquitously expressed proteins (26). Members of this family are involved in a range of biological functions, including lipid binding, neuronal development, serine protease inhibition, and regulation of key signaling pathways (26). RKIP is widely expressed in mammalian tissues, such as the liver, heart, and skeletal muscle (27–29).

**Figure 2:**
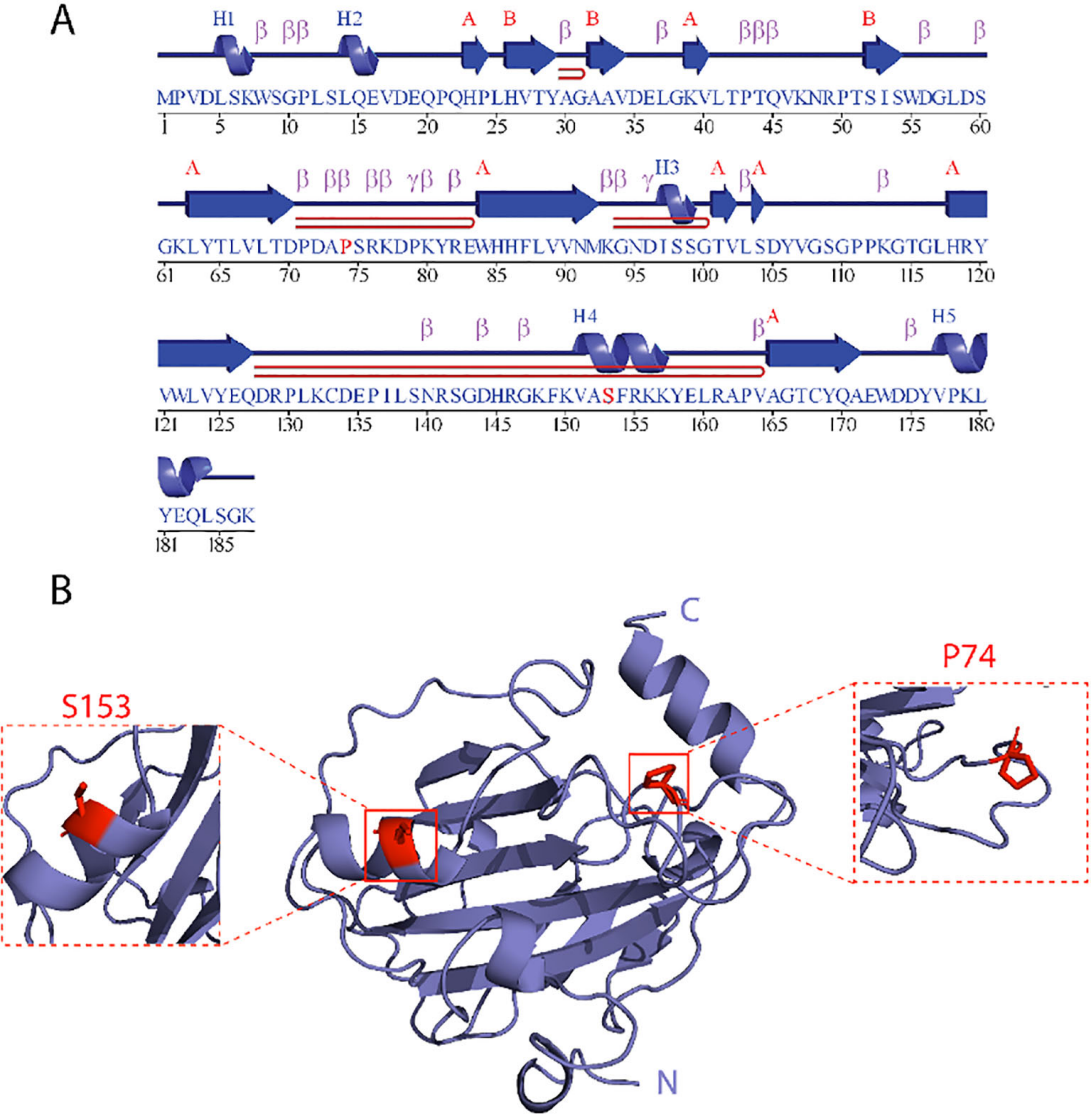
RKIP Structure. **(A)** Primary amino acid sequence of human RKIP, with secondary structure elements. Blue arrows represent β-strands, and purple cylinders indicate α-helices (labeled H1–H5). The sequence is segmented to show the alignment of these structural features along the polypeptide chain. Notably, P74 and S153 are marked in red, emphasizing their importance. P74 is located within the flexible pocket loop (residues 70–85), while S153 is positioned on the α3 helix. **(B)** Tertiary structure of RKIP as a ribbon diagram, with the N- and C-termini labeled. The protein exhibits a central β-sheet core flanked by α-helices, characteristic of the PEBP family fold. Insets zoom in on the locations of S153 and P74, both highlighted in red.

RKIP was first identified as a negative regulator of the MAPK/ERK signaling cascade (also known as the Raf/MEK pathway) by Yeung et al. in 1999 (30). Structurally, RKIP adopts a conserved PEBP family fold composed of an eight-stranded β-sheet flanked by three α-helices (**Figure 2**). Its function is regulated through a dynamic conformational landscape. Phosphorylation at serine 153 (Ser153) by protein kinase C (PKC) triggers a conformational switch from an RKIP-Raf binding state to an RKIP-GRK2 (G protein-coupled receptor kinase 2)-binding conformation. This switch involves partial unfolding of the α3 helix and dimerization of RKIP, which exposes GRK2-binding interfaces (31).

A key regulatory element in this structural transition is proline 74 (P74), located within a flexible loop spanning residues (32–46). P74 governs the equilibrium between three conformational states: the RKIP-Raf-binding ground state, the phosphorylated RKIP-GRK2-binding state, and a high-energy intermediate conformation referred to as RKIP-KIN (47), which is accessible to various kinases. Notably, a single amino acid mutation at P74 to leucine (P74L) increases the conformational flexibility of the loop, stabilizing the RKIP-KIN state. This stabilization enhances Ser153 phosphorylation by approximately threefold compared to the wild-type protein (47).

#### 1.3.2 Yin Yang 1 transcription factor structure-function dynamics

**Figure 3:**
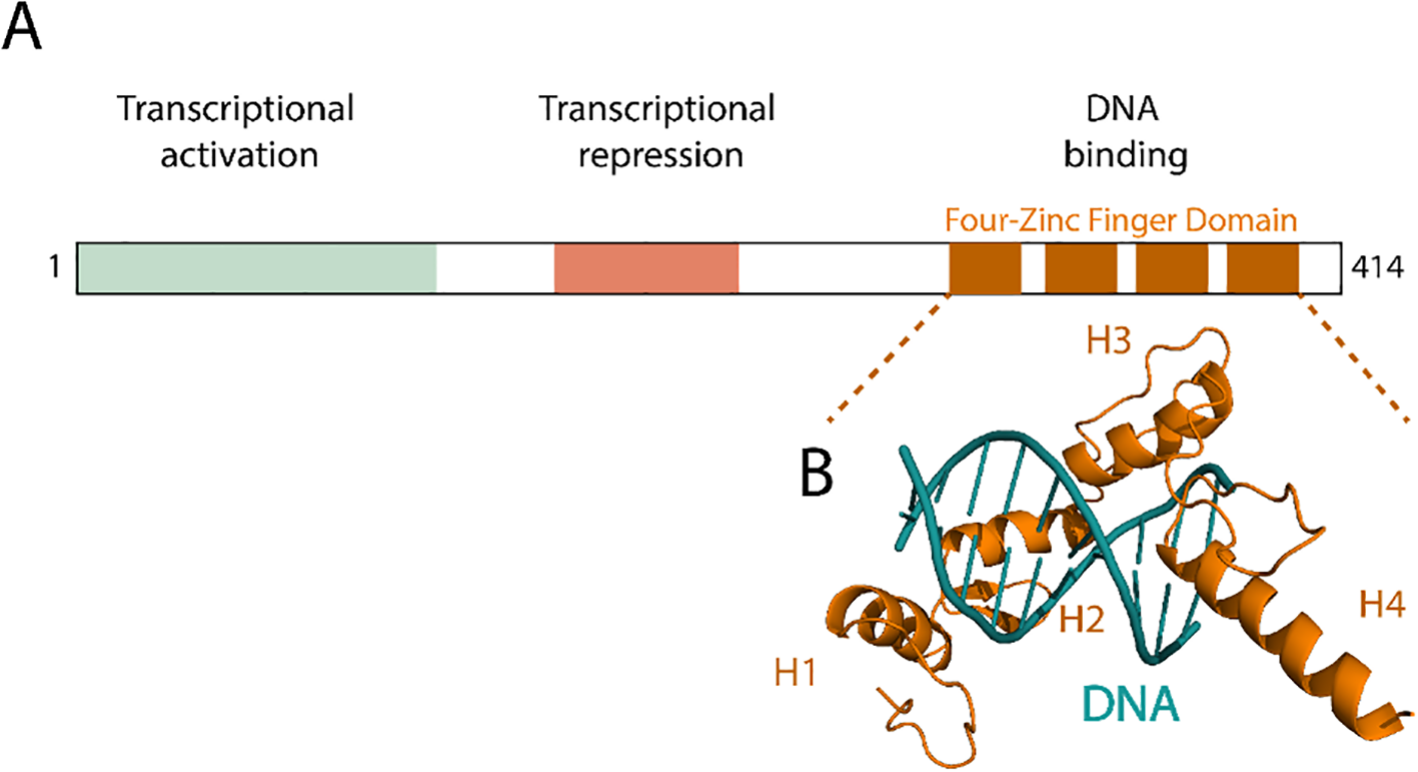
Yin and Yang protein domains. **(A)** Domain architecture of human Yin Yang 1 (YY1), showing an N-terminal transcriptional activation domain, a central repression domain, and a C-terminal four-zinc finger DNA-binding domain. The schematic highlights how these regions correspond to distinct functional roles. **(B)** Four zinc fingers (H1–H4, orange) engaging the DNA helix (teal) with a 5′-CCGCCATNTT-3 motif to mediate sequence-specific binding.

Yin Yang 1 (YY1) is a multifunctional transcription factor and DNA-binding protein (48). It is ubiquitously expressed and plays a broad range of roles in both healthy and diseased tissues (49). Structurally, YY1 comprises 414 amino acids and displays a modular organization that supports its dual functions as a transcriptional activator and repressor (**Figure 3**). The N-terminal region contains a glycine-lysine-rich (GK-rich) domain (amino acids 170–200) and a REPO motif (amino acids 201–226), which are responsible for recruiting histone deacetylases (HDACs) and polycomb repressive complexes (PRC1/2), respectively, thereby mediating transcriptional repression (50). The C-terminal region harbors a C_2_H_2_-type zinc finger DNA-binding domain (amino acids 295–414), which recognizes the consensus DNA motif 5′-CCGCCATNTT-3′ (51) and also facilitates RNA interactions (52).

YY1 function is further regulated by post-translational modifications. Tyrosine phosphorylation at residue Y383, mediated by Src kinases, reduces its ability to bind DNA and RNA, thereby diminishing its repressive activity (53). Additionally, YY1 exhibits structural flexibility that enables homodimerization, a feature that facilitates chromatin looping (54) and contributes to higher-order genome organization. These structural features enable YY1 to function in a context-dependent manner, as a transcriptional activator, repressor, or chromatin organizer, depending on the cellular environment and its interaction with specific cofactors.

#### 1.3.3 Importance of RKIP and YY1 in regulating immune and metabolic pathways

RKIP plays a pivotal role in the regulation of fundamental physiological processes, including cardiac function and neuroactivity (55). Since its initial identification as an inhibitor of the MAPK pathway, RKIP has been implicated in the modulation of diverse cellular signaling cascades governing growth, division, migration, and apoptosis (56–58). Moreover, extensive evidence supports RKIP’s role as a suppressor of tumor metastasis (59, 60) and its potential therapeutic target (60, 61) in cancer.

YY1, by contrast, is a versatile transcription factors that can activate, repress, or initiate transcription depending on the molecular context and its interaction with other DNA-binding regulators (62). YY1 is involved in key processes such as apoptosis induction (59, 62), cellular survival (63), DNA repair (62), and chromatin remodeling (62). It is also a downstream target of nuclear factor kappa-light-chain-enhancer of activated B cells (NF-κB), and has been shown to contribute to immune evasion and chemoresistance in cancer (61, 64).

Importantly, RKIP and YY1 exhibit functionally opposing roles. When YY1 is overexpressed, RKIP is frequently downregulated or absent (65, 66). This inverse relationship is partly mediated by YY1 binding to the RKIP promoter, thereby repressing its transcription. Inhibition of YY1’s DNA-binding activity restores RKIP expression (57).

Conversely, RKIP has also been shown to restrict YY1 expression and activity via inhibition of NF-κB signaling, which results in reduced YY1 transcription and protein levels (49, 67). Through this suppression of YY1, RKIP can alleviate chemoresistance and immune-resistance in cancer cells, thereby sensitizing them to death receptor-mediated apoptosis (63, 68–70).

#### 1.3.4 Importance of RKIP and YY1 in modulation of cellular energetic pathways in metabolic inflammation

Metabolic homeostasis in immune and parenchymal cells is tightly coupled to the dynamic regulation of glycolysis, glutaminolysis, and fatty acid metabolism. These bioenergetic programs are not only essential for energy generation but also dictate immune activation, differentiation, and tissue remodeling during metabolic disease. Within this network, the RKIP and YY1 represent two mechanistically interconnected regulators that coordinate kinase signaling (MAPK, NF-κB) and metabolic sensors (AMPK, mTOR, PGC-1α) to balance catabolic and anabolic energy fluxes. Disruption of this RKIP–YY1 axis underlies the metabolic inflexibility and chronic inflammation observed in obesity, T2D, and CVD.

Under physiological conditions, RKIP maintains metabolic balance by constraining excessive Raf/MEK/ERK and IKK/NF-κB signaling, thus favoring AMPK activation and PGC-1α–driven mitochondrial oxidative phosphorylation (71–74). AMPK acts as a central metabolic switch that promotes fatty acid oxidation, suppresses *de novo* lipogenesis, and limits mTORC1 activity (75, 76). Concurrently, YY1, in complex with PGC-1α, supports transcription of genes involved in mitochondrial biogenesis and oxidative metabolism (77, 78). Together, RKIP and YY1 operate in a balanced and reciprocal manner, RKIP restraining pro-growth kinase cascades while YY1 fine tunes mitochondrial energy output, ensuring efficient nutrient utilization and immune quiescence.

In obesity, chronic nutrient overload and low-grade inflammation perturb this equilibrium. YY1 expression and activity are increased in adipose tissue macrophages and hepatocytes, promoting glycolytic and lipogenic gene expression, including HK2, PFKFB3, FASN, and SCD1 (79). This metabolic reprogramming favors pro-inflammatory and anabolic phenotypes that sustain adipose inflammation. Conversely, RKIP expression is markedly reduced in obese adipose and liver tissues (79), leading to disinhibition of ERK and NF-κB signaling. This results in AMPK suppression and mTORC1 overactivation, further enhancing glycolysis and fatty acid synthesis at the expense of oxidative metabolism (80, 81). In immune cells, such as macrophages and T lymphocytes, this shift drives an M1-like inflammatory polarization and effector T-cell activation, fueling systemic insulin resistance.

During T2D progression, chronic hyperglycemia and lipotoxicity exacerbate YY1-mediated transcriptional control of anabolic pathways. YY1 interacts with mTORC1 to enhance glutaminolysis and amino acid utilization (82). This process supports biosynthetic needs of proliferating immune cells and contributes to β-cell stress and insulin resistance (82). In contrast, RKIP acts as a negative regulator of MAPK–mTOR signaling, and its restoration promotes AMPK activation, enhancing fatty acid oxidation and autophagic clearance of damaged mitochondria (42). Thus, a loss of RKIP function synergizes with YY1 overactivity to reinforce metabolic rigidity and inflammation in insulin-sensitive tissues such as liver, skeletal muscle, and adipose.

In the heart and vasculature, YY1 contributes to maladaptive metabolic remodeling by upregulating glycolytic enzymes and repressing oxidative genes through mTOR and NF-κB–dependent mechanisms (32). YY1 also promotes foam cell formation in macrophages by enhancing fatty acid synthase and scavenger receptor expression (33). In contrast, RKIP exerts cardioprotective actions by attenuating MAPK-driven inflammatory signaling and restoring AMPK activity (34). Through this mechanism, RKIP supports mitochondrial respiration and limits lipid accumulation in cardiomyocytes and vascular macrophages, thereby counteracting YY1-mediated metabolic stress.

**Figure 4:**
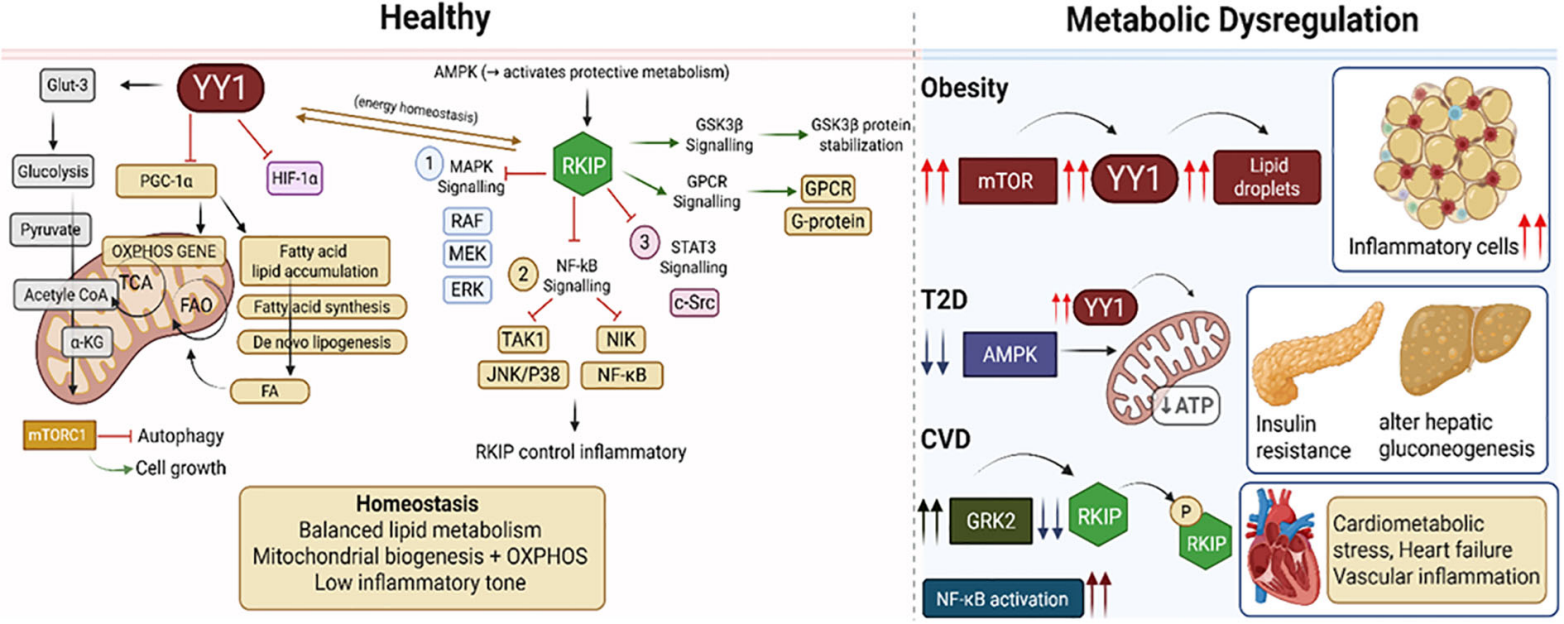
The RKIP–YY1 Axis Orchestrates MAPK, NF-κB, and mTOR Signaling to Balance Inflammation and Metabolism in Health and Disease. Healthy: YY1 supports mitochondrial biogenesis and OXPHOS genes (genes associated with oxidative phosphorylation: a mitochondrial process responsible for producing most of the cell’s ATP), through PGC-1α, while RKIP suppresses MAPK, NF-κB, and STAT3 pathways to maintain low inflammation. AMPK activity promotes energy homeostasis, fatty-acid oxidation, and autophagy. Together, these pathways preserve balanced lipid metabolism and mitochondrial function. Metabolic Dysregulation: In obesity and T2D, increased mTOR and YY1 activity, along with reduced AMPK, drive lipid accumulation, adipose inflammation, mitochondrial stress, insulin resistance and alter hepatic gluconeogenesis. Loss of RKIP function and elevated GRK2 enhance NF-κB signaling, contributing to cardiometabolic disease, heart failure and vascular inflammation.

The RKIP–YY1 interplay converges on three metabolic hubs AMPK, mTOR, and PGC-1α that orchestrate cellular energy flux. RKIP upholds AMPK and PGC-1α activity, sustaining oxidative metabolism, while YY1, under pathological nutrient excess, engages mTORC1 to drive anabolic and inflammatory programs. In healthy states, these pathways maintain a dynamic equilibrium between energy generation and biosynthesis, but in obesity, T2D, and CVD, this balance is lost. **Figure 4** illustrates how RKIP and YY1 oppositely regulate these interconnected routes, glycolysis, glutaminolysis, and fatty acid synthesis, across metabolic tissues, highlighting potential therapeutic points for restoring metabolic harmony.

## 2 Role of RKIP in immune regulation

### 2.1 RKIP in signal transduction pathways (MAPK, NF-κB)

The MAPK cascade is a critical signal transduction pathway activated by diverse extracellular stimuli and involved in regulating cell growth, differentiation, proliferation, and cell survival (34). Within this pathway, rapidly accelerated fibrosarcoma (RAF) kinases transmit signals to extracellular signal-regulated kinases (ERKs) through the RAF-MEK-ERK axis (35).

RKIP negatively regulates this cascade by interfering with RAF-mediated phosphorylation of MEK (36). Overexpression of RKIP suppresses MAPK signaling, whereas its downregulation leads to enhanced pathway activation (36). Mechanistically, RKIP inhibits RAF activation by preventing phosphorylation at key regulatory sites (37–39).

Phosphorylation of RKIP at serine 153 (Ser153) by protein kinase C (PKC) induces its dissociation from RAF, thereby relieving inhibition and enabling activation of the RAF-MEK-ERK cascade (40). This phosphorylation-dependent switch underlies the dynamic regulation of MAPK signaling by RKIP and reflects its role in balancing growth and survival signals. In addition to its role in MAPK signaling, RKIP modulates the NF-κB pathway, which is essential for inflammation and immune responses. NF-κB is activated by pro-inflammatory cytokines in immune cells and regulates genes involved in cell survival (41), immunity, and inflammation. RKIP inhibits NF-κB signaling by blocking the phosphorylation and degradation of IκB, the inhibitory protein that retains NF-κB in the cytoplasm (41–43). By stabilizing IκB, RKIP reduces NF-κB nuclear translocation and transcriptional activity, ultimately suppressing cell survival signaling and tumorigenesis (44).

#### 2.1.1 RKIP in immune system modulation

RKIP plays a critical role in immune system modulation through its interactions with major signaling pathways, particularly NF-κB, and STAT3. These interactions influence the differentiation and activation of macrophages and other immune cell, underscoring RKIP’s importance in regulating inflammatory processes (30, 41, 45, 46, 55, 56, 83–87).

At the cellular level, RKIP exhibits a broad subcellular distribution, localizing to the inner periplasmic membrane, endoplasmic reticulum, cytoplasm, and nucleus (28, 30, 88–92). This widespread localization supports its regulatory roles across multiple immune contexts and signaling environments. Given these functions, RKIP has garnered attention for its involvement in a range of inflammatory and immune-related conditions, including multiple sclerosis, Alzheimer’s disease, rheumatoid arthritis, diabetic nephropathy, systemic inflammatory response syndrome, asthma, allergy, inflammatory bowel disease, colitis, Sjogren’s syndrome, and certain infections such as *Helicobacter pylori* and viral infections (30, 41, 45, 46, 55, 56, 83–87).

In this section, we summarize RKIP’s role in immune modulation by examining its interactions with key signaling molecules, its impact on immune cell function, and its regulatory influence on inflammation, particularly in the context of metabolic diseases such as obesity, diabetes, and CVD.

#### 2.1.2 Mitogen-activated protein kinase

**Figure 5:**
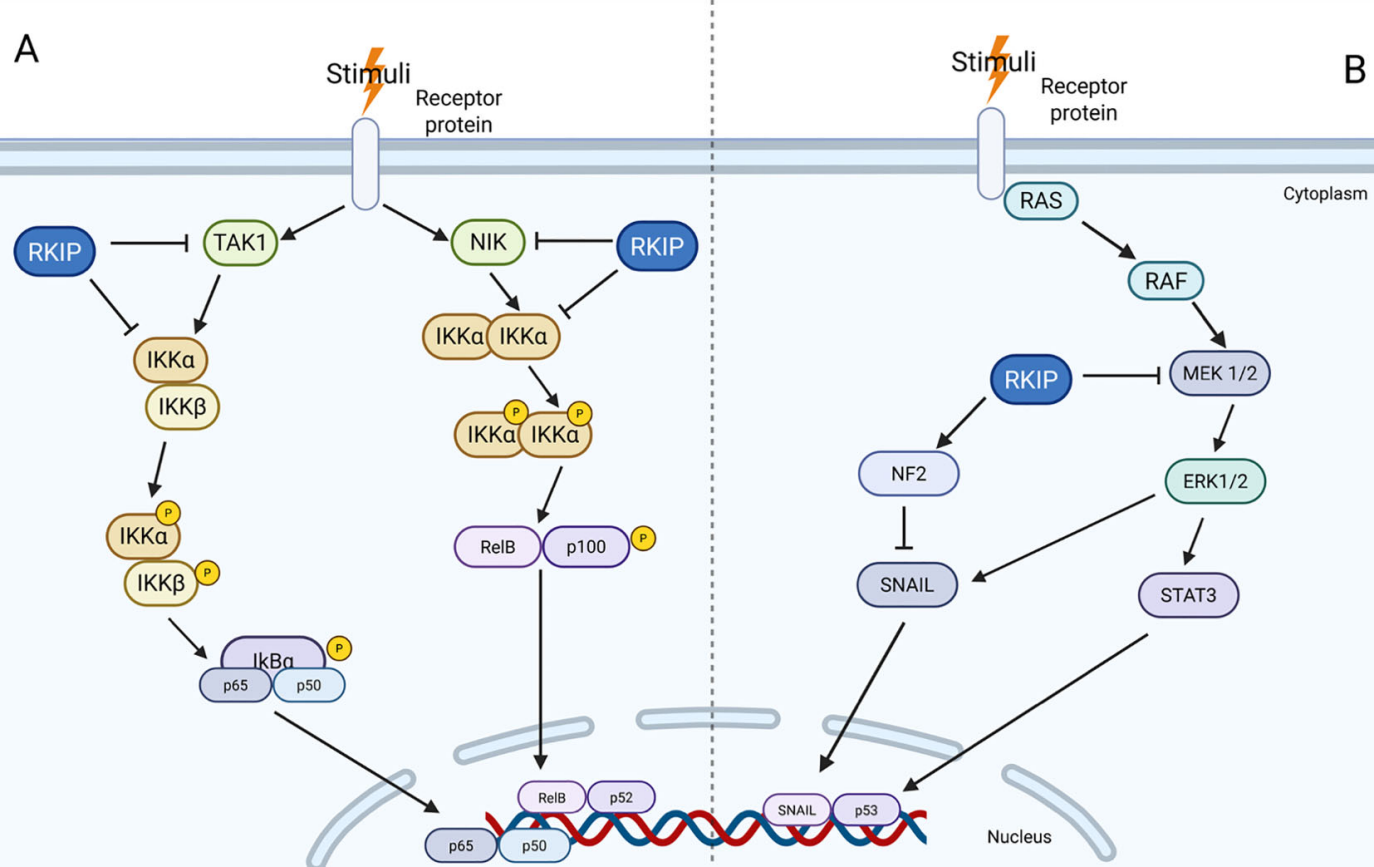
RKIP interaction with NF- κB and MAPK pathways. **(A)** RKIP inhibits key upstream regulators (TAK1 and NIK) to suppress activation of the canonical and non-canonical NF-κB pathways. **(B)** RKIP modulates MAPK signaling by inhibiting RAF–MEK–ERK activation and regulating downstream factors such as STAT3, NF2, and SNAIL.

MAPKs are serine/threonine-specific kinases that mediate cellular responses to a wide range of stimuli, including mitogens, osmotic stress, heat shock, and proinflammatory cytokines. They regulate key biological processes such as cell proliferation, gene expression, differentiation, mitosis, survival, and apoptosis (93). To date, seven MAPK signaling pathways have been identified, several of which are subject to inhibition by RKIP (94) (**Figure 5**). In initial studies, Yeung et al. showed that RKIP inhibits the RAF/MEK/ERK pathway by binding separately to both RAF and MEK, thereby disrupting their interaction. RKIP also regulates MAPK signaling through inhibition of TAK1 (Transforming Growth Factor Beta-Activated Kinase 1) (30, 36, 41), a central kinase that integrates proinflammatory and stress-related stimuli. Upon phosphorylation at Ser153 by protein kinase C (PKC), RKIP dissociates from RAF, allowing RAF to be phosphorylated by p21-activated kinases (PAK) and Src kinases, leading to downstream activation of ERK (36, 95).

TAK1 functions as a master upstream kinase within several MAPK cascades, responding not only to TGF-β but also to signals from Toll-like receptors (TLRs), tumor necrosis factor (TNF), interleukin-1 (IL-1) receptors, and osmotic stress. These stimuli predominantly activate the JNK and p38 MAPKs (96–101). RKIP has been shown to inhibit TAK1-mediated activation of both JNK and p38 (41),, highlighting its broader regulatory role in MAPK signaling beyond the ERK axis. MAPK signaling is also essential for metabolic homeostasis, mediating adaptive responses that maintain normal cellular physiology (11, 102). It intersects with insulin signaling by modulating the activity of insulin receptors. Upon insulin stimulation, the activated insulin receptor recruits adaptor proteins such as Grb2, or insulin receptor substrate (IRS), initiating downstream activation of ERK, JNK and p38 pathways, which are critical for glucose homeostasis (103, 104). Aberrant MAPK signaling is increasingly recognized as a contributor to the development of metabolic disorders, including obesity, diabetes, and hepatic steatosis. While the regulation of MAPK pathways by RKIP has been well-characterized in oncology, its role in modulating MAPK signaling within metabolic disease contexts remains underexplored.

#### 2.1.3 Nuclear factor-κB

NF-κB comprises a family of inducible transcription factor complexes that play a role in regulating immune and inflammatory responses (105, 106). NF-κB signaling occurs via two distinct pathways: the canonical and the noncanonical (or alternative) pathways. Both culminate in the release of an NF-κB dimer, and its translocation to the nucleus, and the regulation of gene transcription (105–107). The canonical NF-κB pathway is broadly responsive to a variety of immune stimuli, including signals from T-cell receptors (TCRs), B-cell receptors (BCRs), TNF receptors (TNFRs), and TLR/IL-1 receptors (IL-1Rs) (108, 109). These signals converge on the IκB kinase (IKK) complex, primarily involving IKKβ and the regulatory subunit NEMO (IKKγ), leading to phosphorylation and subsequent degradation of IκBα (110). The noncanonical NF-κB pathway is activated by a more limited subset of TNFR superfamily members, including CD40, BAFFR (B-cell activating factor receptor), and LTβR (lymphotoxin-β receptor) (111). These stimuli promote the activation of NF-κB-inducing kinase (NIK), which phosphorylates and activates IKKα homodimers. IKKα then induces the processing of the NF-κB p100 precursor into p52, enabling the nuclear translocation of RelB/p52 dimers. These transcription factors regulate gene expression programs involved in lymphoid organogenesis, B cell survival, and adaptive immune responses (107, 112).

RKIP functions as both a scaffold and a negative regulator within the NF-κB signaling network. It has been shown to bind and/or inhibit several key intermediates involved in both the canonical and noncanonical NF-κB pathways, including NIK, IKKα, IKKβ, TAK1, TBK1, PKC, MEK, Act1, and TRAF6 (41, 113). Notably, RKIP directly interacts with NIK, and its expression has been shown to antagonize NIK-induced NF-κB activation, thereby suppressing the noncanonical NF-κB pathway (30, 95, 114–116).

TLRs are critical components of innate immunity, and all human TLRs converge on the NF-κB signaling pathway (11, 117). RKIP has been shown to inhibit NF-κB activation downstream of TLRs signaling (96). In macrophages, RKIP deficiency, either by genetic knockout or RNA silencing, has been associated with significantly reduced expression of IFN-β, IL-6, and TNF following stimulation with poly(I:C), a synthetic analog of viral double-stranded RNA that models TLR3 activation (118). Interestingly, this inhibitory effect was not observed following stimulation with LPS or oligodeoxynucleotides (CpG), which activate TLR4 and TLR9, respectively; cytokine levels remained unchanged in RKIP-deficient cells under these conditions (118). The role of the NF-kB signaling pathway and related mediators has been studied in obesity, T2D, steatotic liver diseases (SLD), and atherosclerosis (119–123). NF-κB regulates the expression of inflammatory mediators that recruit monocytes and promote their differentiation into macrophages, as well as macrophage migration, polarization, and activation (106, 120–131). These responses are driven by various cytokines and chemokines regulated by NF-κB. Other immune cell types, including neutrophils and T cells, are also activated and recruited to sites of inflammation, such as adipose tissue in obesity and insulin resistance, or the liver in steatohepatitis, to sustain immune responses (106, 125–128). Cytokines and pathogen-associated molecular patterns (PAMPs) stimulate cell surface receptors, including TLRs, to initiate signaling cascades that lead to NF-κB activation (106, 132–135). Activation of innate immune receptors such as TLR4 and its adaptor MyD88 can activate NF-κB in response to excess free fatty acids from a high-fat diet (133, 135). The role of RKIP and its inhibitory effect on the NF-κB pathway requires further investigation in the context of metabolic diseases.

#### 2.1.4 G protein-coupled receptor kinase

G protein-coupled receptors (GPCRs) are a large family of membrane-bound receptors that mediate a wide range of physiological functions, including vision, olfaction, and taste, as well as responses to hormones and neurotransmitters (136). Upon ligand binding, GPCRs activate intracellular signaling through two main pathways: the cyclic AMP (cAMP) pathway and the phosphatidylinositol pathway (137). In the cAMP pathway, ligand binding to a GPCR activates its associated G-protein, which in turn stimulates adenylyl cyclase (AC) to convert ATP into cAMP. Elevated cAMP levels activate protein kinase A (PKA) and phosphodiesterases, leading to downstream phosphorylation events.

In the phosphatidylinositol pathway, GPCR activation stimulates phospholipase C (PLC) via G-protein signaling. PLC hydrolyzes phosphatidylinositol 4,5-bisphosphate (PIP_2_) into diacylglycerol (DAG) and inositol 1,4,5-trisphosphate (IP_3_). IP_3_ binds to its receptor on the endoplasmic reticulum (ER), triggering the release of Ca²^+^ into the cytosol. The increase in intracellular Ca²^+^, together with DAG, activates protein kinase C (PKC), which then phosphorylates various cellular targets, including GPCR kinases (GRKs) (138, 139).

Tohgo et al. showed that GRK2 can inhibit GPCR-mediated ERK activation by promoting the assembly of stable scaffolding complexes involving GPCRs, β-arrestins, and MAPK components such as c-Raf, MEK1, and ERK2 (140). These complexes sequester ERK in the cytoplasm, preventing its translocation to the nucleus and thereby suppressing downstream MAPK signaling (140). Under basal conditions, RKIP inhibits RAF activity through direct binding. Upon phosphorylation at Ser153 by PKC, RKIP undergoes a conformational change, dissociates from RAF, and instead binds to GRK2 (141). This switch relieves RAF inhibition and enhances MAPK signaling, while simultaneously inhibiting GRK2, thereby promoting sustained GPCR signaling and further PKC activation.

The stoichiometry of RKIP and its binding partners supports this phosphorylation-dependent regulatory mechanism: RKIP and RAF are present at approximately a 1:1 ratio, whereas RKIP is tenfold more abundant than GRK2 (1:0.1) (141). Upon phosphorylation, dimerized p-RKIP exhibits increased binding affinity for GRK2, effectively shifting its regulatory interaction from RAF to GRK2 (95). Further supporting this model, Robinson et al. identified physical interactions between GRK2, RAF, and RhoGTP downstream of EGFR signaling in vascular tissues, emphasizing GRK2’s scaffolding role in modulating ERK/MAPK pathway activity (142).

GRK2 levels have been shown to rise during left ventricular hypertrophy (LVH) (36, 94–98), and are associated with impaired cardiac contractility (143). In chronic heart failure (HF), elevated GRK2 contributes to multiple pathological processes, including increased cardiac insulin resistance, reduced metabolic flexibility (144, 145), dysregulation of intracellular calcium homeostasis (146), and activation of NF-κB signaling (147). Although the role of RKIP in heart failure has been investigated, its therapeutic potential remains to be fully validated.

#### 2.1.5 Glycogen synthase kinase-3

The α and β isoforms of GSK3 have been extensively studied in the context of metabolic diseases, including obesity, T2D and CVD (148). As a result, numerous GSK3 inhibitors have been developed, many of which are under investigation or in clinical trials for these conditions (148–151). GSK3 plays a pivotal role in regulating a wide array of immune cell types, including macrophages, T cells, dendritic cells, as well as glial cells involved in both immune and non-immune functions. It modulates essential processes such as cell proliferation, differentiation, survival, migration, and macrophage polarization, thereby shaping inflammatory responses (152–154). GSK3 also mediates signaling downstream of several TLRs in human monocytes, particularly influencing TLR-induced inflammatory responses (152, 155). Furthermore, GSK3 has been implicated in the activation of the NOD-like receptor protein 3 (NLRP3) inflammasome, which governs the maturation and secretion of pro-inflammatory cytokines such as IL-1β, a key driver in the development of metabolic syndrome (156, 157). Notably, increased expression of TLRs (especially TLR2 and TLR4), NLRP3, and their ligands has been observed in metabolically relevant tissues, including adipose tissue, skeletal muscle, and the hypothalamus, of individuals with obesity and/or diabetes (158, 159).

Transcriptional regulators such as β-catenin (CTNNB1), SNAIL (Snail Family Transcriptional Repressor 1, SNAI1), and SLUG (Snail Family Transcriptional Repressor 2, SNAI2) are critically involved in cell cycle regulation, epithelial-to-mesenchymal transition (EMT), and inflammatory reprogramming, making them highly relevant in both metabolic and oncogenic contexts (160).

Al-Mulla *et al (*88) demonstrated that reduced RKIP expression enhances oxidative stress, leading to p38 MAPK activation and phosphorylation-dependent inactivation of GSK3-β at threonine 390. This inactivation stabilizes cyclin D and promotes oncogenic signaling pathways involving β-catenin, SNAIL, and SLUG (88).

#### 2.1.6 Signal transducers and activators of transcription 3

STAT3 is a key transcription factor that regulates a broad array of biological processes, including apoptosis, tissue repair, inflammation, hematopoiesis, immune regulation, and adipogenesis (161–163). It is activated by various cytokines, such as IL-6, IL-10, IL-17, oncostatin M, G-CSF, IL-11, EGF, and TNF, all of which are central to the development and progression of inflammatory diseases (164, 165). STAT3 also plays a critical role in natural killer (NK) cell biology by modulating their development, activation, cytotoxicity, and crosstalk with both innate and adaptive immune responses (162, 166). Dysregulated STAT3 activity has been implicated in a variety of pathological conditions, including bone-related diseases (167), atherosclerosis (168), myocardial fibrosis (169), autoimmune and inflammatory disorders (170, 171), neurodegenerative diseases (172), and multiple types of cancers (173).

RKIP negatively regulates STAT3 signaling through multiple mechanisms. Specifically, RKIP inhibits IL-6/JAK1/2-mediated STAT3 activation, as well as c-Src-dependent phosphorylation of STAT3 (59). In prostate and breast cancer patients, high RKIP expression coupled with low STAT3 levels has been associated with improved overall survival, underscoring RKIP’s role as a potential transcriptional suppressor of inflammation and modulator of oncogenic signaling pathways (59). Interestingly, while STAT3 is predominantly associated with pro-inflammatory signaling, it also exerts anti-inflammatory effects under specific conditions - for example, by suppressing NF-κB and MAPK activation through inhibition of Ubc13, an E2 ubiquitin-conjugating enzyme (174). STAT3-deficient macrophages, dendritic cells, and neutrophils display heightened production of TNF-α, IFN-γ, IL-12, and IL-6 following TLR4 stimulation, highlighting STAT3’s dual role in immune modulation (175).

#### 2.1.7 RKIP in metabolic disease and tissue dysfunction

Several studies have demonstrated that RKIP plays multifaceted roles in the development and progression of metabolic and metabolism-related diseases.

#### 2.1.8 Pancreatic function

Early work by Pardo et al. showed that RKIP knockout (KO) mice exhibit increased pancreatic β-cell mass and insulin content, which correlates with improved glucose tolerance (176). Moreover, adult RKIP-KO mice displayed accelerated recovery from streptozotocin-induced diabetes, suggesting that RKIP may negatively regulate pancreatic growth and β cell expansion (176).

#### 2.1.9 Diabetic nephropathy and retinopathy

Rituximab, a CD20-targeting monoclonal antibody used in the treatment of certain autoimmune diseases and cancers, has shown therapeutic potential in preclinical models of diabetic complications. In a rat model of diabetic nephropathy, rituximab upregulated RKIP expression and reduced NF-κB activation, resulting in decreased proteinuria (177). These findings suggest rituximab as a promising candidate for managing diabetic nephropathy (177). In diabetic retinopathy, RKIP suppresses glucose-induced angiogenesis and endothelial–mesenchymal transition in retinal endothelial cells. Overexpression of RKIP reduced cell viability, migration, and tube formation in glucose-stimulated cells (178). Furthermore, lentiviral delivery of RKIP in a rat model of proliferative diabetic retinopathy decreased Müller cell apoptosis by inhibiting p38-MAPK signaling (179).

#### 2.1.10 Stroke and neuroprotection

Using UHPLC-Q-TOF-MS-based metabolomic profiling, Su et al. showed that RKIP overexpression attenuates ischemic brain injury by modulating energy, amino acid, and lipid metabolism, as well as by limiting inflammatory responses (180). Similarly, Gao et al. (2017) reported that pioglitazone enhanced memory performance in diabetic rats, an effect partially attributed to ERK1/2 activation and downregulation of hippocampal RKIP expression (181).

#### 2.1.11 Inflammation and autoimmunity

RKIP Overexpression suppresses the release of chemokines and cytokines, thereby reducing autoimmune inflammation, as observed in models of systemic lupus erythematosus (SLE) (182). Conversely, RKIP has also been shown to enhance IL-17R signaling, amplifying MAPK and NF-κB pathway activation during inflammatory responses (114). Mechanistically, RKIP interacts with ASC (apoptosis-associated speck-like protein containing a CARD), a central adaptor protein in inflammasome assembly. This interaction prevents the formation of inflammasomes such as NLRP1, NLRP3, and NLRC4, thereby inhibiting IL-1β production. Notably, RKIP expression is downregulated in patients with T2D and gout, implicating it in inflammasome-associated pathologies (183).

#### 2.1.12 Cardiometabolic disease and GLP-1 signaling

A 2024 study demonstrated that the GLP-1 receptor agonist Semaglutide reduces cardiac inflammation and fibrosis in diabetic mice by restoring RKIP expression and suppressing the TBK1–NF-κB signaling axis. These cardioprotective effects were mediated through Sirt3 and cAMP/PKA pathways and were markedly diminished in RKIP-deficient mice (184), underscoring the critical role of RKIP in GLP-1 induced cardiac benefits.

#### 2.1.13 Hepatic necroptosis and cancer

In obese db/db mice and high-fat diet-fed models, AAV-induced hepatic necroptosis was shown to be RKIP-dependent, indicating a possible role for RKIP in the progression of obesity-associated liver cancer (185).

#### 2.1.14 Renal fibrosis and AR signaling

Yao et al. showed that icariin, a bioactive flavonoid compound, attenuates renal fibrosis and endothelial mesenchymal transition in diabetic nephropathy by upregulating RKIP expression and inhibiting the AR/RKIP/MEK/ERK signaling pathway (186).

#### 2.1.15 Population-level proteomics

A large-scale 2025 proteomic analysis involving over 53,000 participants from the UK Biobank found that plasma RKIP levels were inversely associated with obesity, but positively associated with hypertension, coronary atherosclerosis, stroke, myocardial infarction, metabolic dysfunction-associated steatotic liver disease (MASLD), and T2D without complications. No significant association was observed between RKIP levels and T1D (187).

## 3 Role of YY1 in immune regulation

### 3.1 Molecular mechanisms of YY1

YY1 is a multifunctional transcription factor with essential roles in development, immune regulation, and metabolism (188). Depending on the promoter context, cellular environment, and cofactor recruitment, YY1 functions as either a transcriptional activator or repressor (52). This dual capacity is mediated through its interactions with chromatin remodeling and transcriptional co-regulatory complexes, including histone deacetylases (HDAC), histone acetyltransferases (HAT), and Polycomb group (PcG) proteins (189), that collectively shape chromatin accessibility and drive context specific gene expression programs. Beyond its transcriptional activity, YY1 also plays a structural role in genome organization. It facilitates chromatin looping and enhancer-promoter interactions, enabling the coordinated expression of lineage-specific and stimulus-responsive genes (190, 191). This architectural function is particularly critical in metabolically active and immune-responsive cells, where precise and rapid transcriptional adaptation is essential for homeostasis.

### 3.2 YY1 acts as a regulatory hub linking metabolic control with immune signaling

In tissues such as adipose, liver, and skeletal muscle, YY1 regulates pathways involved in mitochondrial biogenesis, oxidative phosphorylation, and lipid metabolism (192, 193). These effects are mediated through interactions with coactivators such as PGC-1α and nuclear hormone receptors, ensuring integration of energy homeostasis with nutrient sensing (193). These metabolic pathways, in turn, influence immune cell behavior, particularly in nutrient-sensitive and inflammatory environments.YY1 also governs the development and function of both innate and adaptive immune cells. It is essential for B cell maturation, T cell lineage specification, and macrophage polarization (194, 195). Furthermore, it modulates the expression of inflammatory mediators such as STAT family members, NF-κB, IL-6, and TNF, positioning it as a central regulator of immune activation (67, 196, 197). Concurrently, YY1 coordinates the expression of genes involved in glucose and lipid metabolism, underscoring its integrative role in linking immune and metabolic networks (194, 198). Under conditions of chronic nutrient excess, such as obesity and T2D, YY1 expression and function become dysregulated, contributing to elevated inflammation, impaired insulin signaling, and disrupted lipid homeostasis. These findings highlight YY1 as a key mediator in the pathogenesis of immune-metabolic disorders.

### 3.3 YY1 in immune system modulation

YY1 is a pivotal regulator of immune cell development and function, exerting context-dependent roles across T cells, B cells, and macrophages. In T cells, YY1 is indispensable for early thymocyte development and lineage specification. It facilitates the transition from double-negative (DN)1 to DN2 thymocytes, independent of its PcG-mediated chromatin remodeling activity, although PcG functions remain critical for thymocyte survival (199, 200). YY1 also supports the development of invariant natural killer T (iNKT) cells by enhancing cell survival and upregulating promyelocytic leukemia zinc finger (PLZF), a lineage-specific transcription factor. Loss of YY1 in thymocytes leads to a marked reduction in iNKT cells, predominantly due to increased apoptosis (201).

YY1 further governs the differentiation of mature T cell subsets. In CD4^+^ T helper 2 (Th2) cells, YY1 cooperates with GATA3 to promote chromatic remodeling at the Th2 cytokine locus, enhancing cytokine production (202). In regulatory T cells (Tregs), YY1 maintains Foxp3 expression, essential for Treg function; its disruption impairs immunosuppressive capacity and exacerbates inflammatory responses (203). Moreover, YY1 contributes to T cell exhaustion by upregulating inhibitory receptors such as PD-1, Tim-3, and Lag-3, while downregulating IL-2 and IFN-γ (204).

In B cells, YY1 is essential for immunoglobulin gene rearrangement and the germinal center reaction (195). It regulates the expression of activation-induced cytidine deaminase (AID), a key enzyme in somatic hypermutation (SHM) and class-switch recombination (CSR), both fundamental to antibody diversity (205, 206). Deletion of YY1 in mouse B cells leads to impaired SHM and CSR. Despite preserved proliferation and transcription, YY1-deficient B cells exhibit defective antibody maturation, likely due to impaired interaction with AID, as demonstrated by Liu et al. and Zaprazna et al (205, 207).

In macrophages, YY1 orchestrates polarization and functional reprogramming. In the tumor microenvironment, particularly in prostate cancer, YY1 is enriched in M2-like macrophages and promotes IL-6 expression, contributing to tumor progression (196). In atherosclerosis, YY1 is upregulated in macrophages exposed to oxidized LDL (oxLDL), where it drives foam cell formation, lipid accumulation, and inflammatory gene expression (33).

Taken together, YY1 plays multifaceted and context-dependent roles in immune modulation. It regulates key processes such as immune differentiation, inflammation, lipid metabolism, and autophagy, critical pathways in the pathogenesis of cancer, atherosclerosis, and metabolic disorders. While YY1 can promote pathological responses such as lipid accumulation and tumor-supportive polarization, it also sustains immune homeostasis and cell survival under stress (194, 208, 209). Its dual function as a transcriptional activator and repressor underscores YY1’s potential as a complex yet promising therapeutic target in immune-mediated diseases (**Table 1**).

**Table 1:** RKIP in metabolic disease and tissue dysfunction.

Metabolic model (cell line and/or animal strain)	Main target in the model (what was modulated)	Therapy used (compound, genetic tool, biologic)	Posology (dose/concentration, route, schedule/duration)	Main biological effects	Effects on RKIP or YY1 (expression/activity, direction, mechanism)	Side effects/safety signals (if reported)	Reference
C57BL/6 mice (Rkip-/-), streptozotocin-induced diabetes	Pancreas, beta cells	Rkip-/-, streptozotocin	IP injection of STZ 100mg/kg	improved glucose homeostasis by IPGTT, ↑ plasma insulin, ↑ pancreas size, beta cell mass, insulin content at steady state, ↑ reversal of STZ-induced diabetes in Rkip-/- mice	NA	Rkip highly expressed in pancreatic islets, mostly localized to beta cells, also expressed in CK19+ ductal cells	Pardo, F. N. et al. The role of Raf-1 kinase inhibitor protein in the regulation of pancreatic beta cell proliferation in mice. Diabetologia 55, 3331–3340 (2012) (176).
Sprague-Dawley (SD) rats, STZ-induced diabetes and HFD	Kidney, diabetic nephropathy	Rituximab	58 mg/kg RTX administered to tail vein once per week for 4 weeks	↑ urine protein and glycemia with STZ and HFD, ↓urine protein with RTX. Worsened kidney histology with STZ and HFD, improved with RTX.	↓RKIP protein expression in the kidney with STZ and HFD compared to healthy control. ↓ RKIP protein expression in the kidney of RTX treated mice compared healthy control group, ↑ compared to STZ and HFD group. RKIP expression negatively correlated to NFkB expression		Li, L. et al. Rituximab regulates the expression of the Raf kinase inhibitor protein via NF-κB in renal tissue of rats with diabetic nephropathy. Genet Mol Res 12, 2973–2981 (2013) (177).
Human retinal capillary endothelial cells (HRCECs)	Response to glucose loading	Knockdown and lentiviral overexpression of RKIP	Glucose at multiple concentrations and timepoints	RKIP-KD ↑ HRCEC viability, migration and angiogenesis in response to high glucose, comapred to WT cells. RKIP-OE ↓ HRCEC viability, migration and angiogenesis in response to high glucose, comapred to WT cells.	No effect of glucose treatment on RKIP expression	NA	Feng, L., Zhang, C., Liu, G. & Wang, F. RKIP negatively regulates the glucose induced angiogenesis and endothelial-mesenchymal transition in retinal endothelial cells. Exp Eye Res 189, 107851 (2019). _181_
STZ-induced diabetes in rats; Rat Müller cells (rMC-1 cell line)	Diabetic retinal neurodegeneration	Lentiviral overexpression of RKIP	IP injection of STZ 55 mg/kg	High glucose ↑ rMC-1 cell apoptosis, RKIP-OE ↓ apoptosis. STZ-induced diabetes ↑ expression of p38-MAPK, GFAP and CASP3. Intravitreal lentiviral overexpression of RKIP reversed these increases.	NA	NA	Wu, C. et al. Protective Effect of Raf-1 Kinase Inhibitory Protein on Diabetic Retinal Neurodegeneration through P38-MAPK Pathway. Curr Eye Res 47, 135–142 (2022) (179).
SD rats	Ischemic stroke (brain)	Focal middle cerebral artery occlusion w/o reperfusion, lentiviral overexpression of RKIP in the cerebral cortex	NA	RKIP OE ↓ necrotic area after ischemic stroke modelling compared to WT. Amino acid, lipid and energy metabolism are associated with RKIP OE being protective.	RKIP expression ↓ with ischemic stroke model.	NA	Su, L., Zhao, H., Zhang, X., Lou, Z. & Dong, X. UHPLC-Q-TOF-MS based serum metabonomics revealed the metabolic perturbations of ischemic stroke and the protective effect of RKIP in rat models. Mol. BioSyst. 12, 1831–1841 (2016) (180).

↑, Higher effects; ↓ Lower effects.

### 3.4 YY1 and metabolic diseases and tissue dysfunction

Emerging evidence implicates YY1 as a key pathogenic factor in the development of metabolic disorders, including insulin resistance, obesity, T2D, and metabolic dysfunction-associated steatotic liver disease (MASLD) (79, 210). YY1 exerts its effects by integrating inflammatory and metabolic signals across metabolically active tissues, namely, adipose tissue, liver, and skeletal muscle. This position is YY1 at the critical intersection of immune and metabolic homeostasis.

#### 3.4.1 Liver disease

In the liver, YY1 contributes to the development and progression of MASLD, including its transition to steatohepatitis and cirrhosis (211). It regulates key pathways involved in lipid metabolism, glucose metabolism, and bile acid synthesis, critical processes in the pathogenesis of liver disease (79). YY1 upregulation promotes lipogenesis and hepatic steatosis by activating genes responsible for lipid biosynthesis. Pan et al. demonstrated that silencing YY1 suppresses hepatic lipogenic genes, particularly *SREBP1* (sterol regulatory element-binding protein 1) and *CHREBP* (carbohydrate-responsive element-binding protein) (79). In addition to its metabolic functions, YY1 also modulates liver immune responses, potentially influencing liver allograft rejection (212), antiviral immunity, and tumorigenesis in conditions such as hepatitis B and hepatocellular carcinoma (213).

#### 3.4.2 Muscle and insulin sensitivity

In skeletal muscle, YY1 regulates muscle regeneration by modulating metabolic reprogramming in satellite (muscle stem) cells (214). It plays a critical role in the metabolic shift between glycolysis and mitochondrial oxidation (193). YY1 also governs mitochondrial biogenesis and oxidative metabolism through interactions with PGC-1α, mTOR, and other transcriptional coactivators (192). Proper mitochondrial function is essential for maintaining insulin sensitivity and systemic energy balance (215). YY1 knockdown reduces the expression of mitochondrial genes and impairs cellular respiration, while mTOR inhibition disrupts YY1–PGC-1α interactions (192). These findings suggest that YY1 dysregulation in muscle impairs fatty acid oxidation and mitochondrial efficiency, contributing to glucose intolerance and systemic metabolic dysfunction.

#### 3.4.3 Adipose dysfunction

In adipose tissue, YY1 orchestrates adipocyte differentiation, lipid metabolism, and inflammatory tone (216). In 3T3-L1 preadipocytes, YY1 interacts physically with PPARγ and C/EBPβ, attenuating PPARγ transcriptional activity. YY1 overexpression disrupts C/EBPβ binding to the PPARγ promoter, leading to downregulation of PPARγ expression and suppression of adipogenesis (217). In brown adipose tissue, YY1 also regulates thermogenesis through dual roles: as a transcriptional activator, it promotes canonical thermogenic and mitochondrial energy expenditure pathways; conversely, as a repressor, it inhibits the expression of genes involved in energy dissipation (218).

In summary, YY1 emerges as a central regulator of metabolic homeostasis, integrating nutrient availability, hormonal signals, and inflammatory stress across key metabolic tissues. Its dysregulation contributes to hallmark features of metabolic disease, including insulin resistance, hepatic steatosis, and mitochondrial dysfunction, positioning YY1 as a promising therapeutic target in metabolic disorders (**Table 2**).

**Table 2:** YY1 in metabolic diseases and tissue dysfunction.

Metabolic model (cell line and/or animal strain; note species)	Main target in the model (what was modulated)	Therapy used (compound, genetic tool, biologic)	Posology (dose/concentration, route, schedule/duration)	Main biological effects (succinct, quantitative when possible)	Effects on YY1 (expression/activity, direction, mechanism)	Side effects/safety signals (if reported)	Reference
HepG2 Cells, Human 293T Cells	Liver diseases, NAFLD	1- Lentiviruses. 2- Sequabrene. 3- Puromycin.	Lentiviruses were produced in 293T cells and used to transduce HepG2 cells. Sequabrene (8 g/ml). Puromycin (0.05 and 1 µg/ml).	Knockdown of Yin Yang 1 (YY1) significantly promotes Lipid Metabolism	YY1 knockdown affects hepatic lipid metabolism by ↓CHREBP, ↓SREBF2, ↓FABP1, ↓SCD, and ELOVL6, highlighting the regulatory mechanisms governing lipid homeostasis.	NA	Pan, G. et al. Multifaceted regulation of hepatic lipid metabolism by YY1. Life Sci Alliance 4, e202000928 (2021) (79)
Male Lewis rat, Brown Norway (BN) rats.	Rat liver transplantation model. Dendritic cells (DCs) to overexpress YY1	1-Enrofloxacin. 2- Carprofen.	Enrofloxacin (10 mg/kg) IM injection once daily for two consecutive days. Carprofen (5 mg/kg) SC injection twice daily for three consecutive days.	Inflammatory phenotypes and acute rejection in liver transplantation.	↑ YY1 expression in inflammatory cells of allografts on days 5 and 10 post-transplant. ↑ expression of YY1 in dendritic cells promotes their activation and maturation.	Complications of Liver Transplantation: Acute rejection Administration of immunosuppressive drugs causes side effects Ischemia-Reperfusion Injury (IRI) In rat liver transplantation model, mice in the allogeneic group died within 15 days	Chen, Y. et al. The novel role of Yin Yang 1 in acute rejection of liver allografts through activation of dendritic cells. Front. Immunol. 16, (2025) (212).
Yy1f/f and C57BL/10 ScSn DMDmdx (mdx) mouse strains. Mouse C2C12 myoblast cells and 293T cells were used.	Adult skeletal stem cells (MuSCs). Mouse model of Duchenne muscular dystrophy (DMD)	1- Amoxifen. 2- Maraviroc. 3- EdU.	Amoxifen IP injection for 5 consecutive days. Maraviroc IP injection at a dose of (2 mg/kg). EdU IP injection (5 mg) 3 consecutive days.	YY1 Deletion in MuSCs leads to muscle dystrophy in mdx mice. Pharmacological blockade of the CCL5/CCR5 axis with Maraviroc effectively mitigated muscle dystrophy and improved muscle performance in dKO mice.	YY1 Deletion in MuSCs leads to ↑ fibrosis, ↑ inflammatory markers, ↓ number of macrophages. The dKO mice showed ↓ body and muscle weight, ↓ muscle thickness. Blockade of the CCL5/CCR5 axis with Maraviroc mitigated muscle dystrophy and improved muscle performance in dKO mice. This led to ↑ muscle regeneration. ↓ fibrosis, ↓ numbers of FAPs and MPs in dKO muscle niche, along with ↓ TGFβ1 expression.	NA	Li, Y. et al. Skeletal muscle stem cells modulate niche function in Duchenne muscular dystrophy mouse through YY1-CCL5 axis. Nat Commun 16, 1324 (2025) (214)
3T3-L1 preadipocytes	Adipose tissue model for adipocyte differentiation	1-IGF-1. 2-YY1 stealth siRNA.	IGF-1 (5, 10, and 20 nM). YY1 stealth siRNA (10 pmol/ml).	FBS suppressed CHOP-10 expression. IGF-1 downregulates CHOP-10	The knockdown of YY1 expression with YY1 siRNA ↑ the expression of CHOP-10, inhibiting adipocyte differentiation.	NA	Huang, H.-Y. et al. Transcription factor YY1 promotes adipogenesis via inhibiting CHOP-10 expression. Biochemical and Biophysical Research Communications 375, 496–500 (2008) (216).
3T3-L1 preadipocytes	Adipose tissue model for adipogenesis, the differentiation of fibroblast-like mesenchymal stem cells into adipocytes.	1- Hormonal cocktail consisting of 3-isobutyl-1-methylxanthine, dexamethasone, and insulin. 2-Rosiglitazone, a known PPARγ agonist.	Differentiation Medium (MDI): consists of 0.5 mM 3-isobutyl-1-methylxanthine, (1 µM) dexamethasone, and (10 µg/mL) insulin in 10% FBS. Hormonal Stimulation: Cells exposed to MDI for 48 hours with DMEM (10 µg/mL) insulin. 3T3-L1 cells were transfected with plasmids or shRNA. For luciferase reporter assays, cells were co-transfected with reporter plasmids and expression plasmids for 36 hours. Rosiglitazone: cells were cultured with (1 µM) for 16 hours after 24 hours of transfection.	YY1 overexpression inhibited adipocyte differentiation, while YY1 knockdown increased adipocyte differentiation. ↓ lipid droplet accumulated with YY1 overexpression.	YY1 mRNA decreased after MDI hormonal induction and increased from day 3 onwards, reaching a maximum on day 8. Protein levels showed ↓ on day 1 and then continuously increased. YY1 repressed the activity of adipogenic transcriptional factors. YY1 directly ↓ PPARγ transcriptional activity.	NA	Han, Y. et al. Yin Yang 1 is a multi-functional regulator of adipocyte differentiation in 3T3-L1 cells. Molecular and Cellular Endocrinology 413, 217–227 (2015) (217)
YY1-Ucp1Cre and YY1-AdipoCre mice. C57BL/6 mice. Brown fat cell line De2.3.	Adipose tissue -Brown adipose tissue (BAT) model	1- Forskolin. 2- Adipogenic cocktail (IBMX, Dexamethasone, Rosiglitazone, Insulin, T3).	Forskolin was used in brown fat cell line De2.3. Adipogenic cocktail (0.5 mM IBMX, 1 µM Dexamethasone, 1 µM Rosiglitazone, 0.02 µM Insulin, and 1 nM T3)	Mice lacking YY1 in adipose tissue were strongly protected against diet-induced obesity. YY1-deficient mice exhibited increased energy expenditure and oxygen consumption in beige and white fat depots.	YY1 plays an antagonistic role in controlling thermogenic gene expression in BAT. YY1 deficiency in BAT led to ↑ expression of FGF21, BMP8b, GDF15, Angptl6, Neuromedin B & Nesfatin YY1 ↓ expression of Bmp8b and Gdf15 and maintains the repressive epigenetic mark H3K27 trimethylation.	NA	Verdeguer, F. et al. Brown Adipose YY1 Deficiency Activates Expression of Secreted Proteins Linked to Energy Expenditure and Prevents Diet-Induced Obesity. Mol Cell Biol 36, 184–196 (2015 (218)
BV2 microglial cells and HEK293T cells.	Neuroinflammation model.	1- LPS. 2- PDTC.	BV2 cells were stimulated with (1 µg/mL) LPS for 0, 0.5, 1, 4, 6, and 12 hours. BV2 cells were treated with (10 µM) PDTC for 30 minutes before LPS stimulation.	LPS stimulation significantly ↑ expression of IL-1β, IL-6, TNF-α, and iNOS mRNA in BV2 cells. Knockdown of YY1 inhibited LPS-induced activation of NF-κB signaling and IL-6 expression in BV2 cells.	YY1 was upregulated in BV2 microglial cells stimulated with LPS, an effect dependent on transactivation function of NF-κB. YY1, ↓ H3K27ac modification on the IL-6 promoter by interacting with HDAC1	NA	Zhang, X.-C. et al. YY1 promotes IL-6 expression in LPS-stimulated BV2 microglial cells by interacting with p65 to promote transcriptional activation of IL-6. Biochem Biophys Res Commun 502, 269–275 (2018) (219)
Murine macrophage cell line, RAW 264.7.	Macrophages- lung cancer.	1-LPS. 2-Live Pseudomonas aeruginosa 103 (PA103). 3- plasmid (pCMV-YY1)	LPS (1µg/ml) to stimulate macrophages. Live Pseudomonas aeruginosa 103 was inoculated on cell at a concentration of (5x10^5^ CFU)for 5 hours. A plasmid encoding human YY1 used in RAW 264.7 cells. Transfection was performed using GenePORTER II, and each transfection was normalized with pCMV plasmid. Cells were treated with LPS for 5 hours for luciferase assays.	Overexpression of YY1 enhanced COX-2 transcriptional activity in macrophages treated with LPS or Pseudomonas aeruginosa. YY1 overexpression enhanced PGD2 production in LPS-treated macrophages, indicating increased functional COX-2 protein.	LPS treatment disrupted the intrinsic interaction between YY1 and p300 but did not affect the interaction with HDAC1/2. This disruption of YY1-p300 interaction contributes to COX-2 expression.	NA	Joo, M. et al. Yin Yang 1 enhances cyclooxygenase-2 gene expression in macrophages. Am J Physiol Lung Cell Mol Physiol 292, L1219-1226 (2007) (220).
Peripheral blood mononuclear cells (PBMCs) were isolated from 35 rheumatoid arthritis (RA) patients (5 men, 30 women, aged 35–69 years) and 30 osteoarthritis (OA) patients, along with 25 healthy donors (HD). 293T cells were used for luciferase assays. Male DBA/1J mice were used to establish a collagen-induced arthritis (CIA) model.	Rheumatoid arthritis	1- Lentiviruses.	(CIA Mice): Mice were treated with lentiviruses (LV-YY1-shRNA, LV-YY1, or LV-NC) via tail intravenous injection at a dose of (4 × 10^7^)TU/mouse. PBMCs from RA patients: Infected with lentivirus in the presence of Dynabeads Human T-activator CD3/CD28 and IL-2 (10 IU/ml) for 48 hours. 293T cells: Transfected with IL-6 promoter plasmids and then infected with lentivirus (LV-YY1-shRNA, LV-YY1, or LV-NC) at an MOI with (5 µg/ml) of polybrene.	Blocking YY1 action with YY1 shRNA lentivirus ameliorated disease progression in CIA mice. Histological examination showed ↓ leukocyte infiltration, cartilage destruction, and bone erosion in treated mice. YY1 blockade significantly ↓ the inflammatory score in CIA mice and inhibited T cell proliferation in response to CII antigen. Blocking YY1 led to ↓ IL-6 production and JAK/Stat in PBMCs from RA patients. YY1 blockade led to ↓ Th17 population in CIA mice.	YY1 was found to be overexpressed in RA patients and CIA mice (spleen and lymph node) compared to healthy controls. YY1 positively regulated IL-6 transcription.	NA	Lin, J. et al. A critical role of transcription factor YY1 in rheumatoid arthritis by regulation of interleukin-6. J Autoimmun 77, 67–75 (2017) (221)
Female C57BL/6J mice, were used to establish an experimental autoimmune uveitis (EAU) model. Human microglia clone 3 (HMC3) cells and primary microglia.	autoimmune uveitis (EAU) model	1- Rotenone. 2- Sodium Dichloroacetate (DCA). 3- A-485. 4- Lentivirus carrying FLAG-tagged YY1 WT or YY1 K183R mutants. 5- LPS. 6- IFN-γ.	Rotenone: 1.5 mg kg^−^¹ IP injection DCAP: injection 5 consecutive days. A-485: 200 µM, 1 µl eye^−^¹ IV injected on day 9. LPS+IFN-γ (Stimulation): *In vitro*: 1 µg mL^−^¹ LPS and 500 ng mL^−^¹ IFN-γ for 24 hours. Lentivirus Infection: MOI of 30 for 24 hours.	Rotenone enhanced inflammation & retinal detachment, while DCA alleviated EAU. Rotenone promoted microglial activation, while DCA decreased it. LPS+IFN-γ strengthened microglial migration and proliferation. YY1 de-lactylation alleviated microglial migration and proliferation. Rotenone enhanced the expression of inflammatory cytokines (iNOS, COX-2, TNF-α), where DCA improved this effect.	YY1 Lactylation Levels: Increased in retinal microglia during EAU. Rotenone significantly enhanced YY1 lactylation. LPS+IFN-γ stimulation ↑YY1 lactylation *in vitro*. YY1 K183R mutation reduced YY1 lactylation levels. p300 overexpression ↑YY1 lactylation, while p300 inhibition (A-485) downregulated it. YY1 Expression unchanged in EAU compared to control. YY1 lactylation promoted microglial dysfunction by ↑ inflammatory cytokine secretion, STAT3, CCL5, IRF1, IDO1, and SEMA4D.	NA	Huang, J. et al. YY1 Lactylation Aggravates Autoimmune Uveitis by Enhancing Microglial Functions via Inflammatory Genes. Advanced Science 11, 2308031 (2024) (222)

↑, Higher effects; ↓ Lower effects.

### 3.5 Role of RKIP and YY1 in non-conventional and tissue-specific immune cells

Although macrophages, T cells, and dendritic cells are well-characterized contributors to immunometabolic dysfunction, increasing evidence highlights the importance of historically “neglected” immune cell subsets—including myeloid-derived suppressor cells (MDSCs), mast cells, neutrophils, innate lymphoid cells (ILCs), eosinophils, basophils, B cells, and tissue-resident macrophages such as microglia in shaping metabolic inflammation and tissue homeostasis. In this context, RKIP and YY1emerge as pivotal regulators of intracellular signaling networks that integrate inflammatory and metabolic cues across these diverse immune cell types.

#### 3.5.1 MDSCs

MDSCs expand in obesity, T2D, and cardiovascular diseases, where they suppress adaptive immune responses yet paradoxically promote low-grade inflammation through cytokine and reactive oxygen species (ROS) release. YY1 supports MDSC survival and differentiation via activation of STAT3 and mTOR signaling, enhancing their glycolytic and lipid metabolic capacity (223, 224). In contrast, RKIP restrains MDSC expansion by inhibiting NF-κB and MAPK cascades, which are essential for myeloid progenitor activation under chronic metabolic stress.

#### 3.5.2 Mast cells, eosinophils, and basophils

Mast cells and basophils contribute to adipose tissue inflammation by releasing histamine, TNF-α, and IL-6, which modulate insulin sensitivity and vascular tone. YY1 enhances its activation and cytokine production through NFAT and mTOR-dependent transcriptional programs, while RKIP acts as a negative regulator of MAPK-mediated degranulation (35, 225). Conversely, eosinophils play protective roles in lean adipose tissue, supporting M2 macrophage polarization via IL-4 secretion. YY1 controls eosinophil differentiation and mitochondrial metabolism, whereas RKIP helps preserve eosinophil quiescence by dampening ERK and NF-κB signaling (226–228).

#### 3.5.3 Neutrophils

Neutrophil infiltration marks early adipose tissue inflammation and contributes to insulin resistance through elastase and NETosis. YY1 regulates neutrophil glycolysis and survival by targeting genes involved in mitochondrial biogenesis and autophagy, promoting a pro-inflammatory phenotype (229). RKIP, in contrast, suppresses excessive neutrophil activation and ERK-dependent NET formation, preserving tissue homeostasis during early metabolic stress.

#### 3.5.4 Innate lymphoid cells

ILCs act as key modulators of barrier integrity and adipose remodeling. YY1 is required for ILC2 differentiation and cytokine production (IL-5, IL-13), promoting thermogenesis and beiging in subcutaneous fat (230, 231). Although data on RKIP in ILCs remain limited, its suppression of MAPK and NF-κB suggests an indirect role in modulating ILC activation through upstream inflammatory signaling. The balance of YY1-mediated activation and RKIP-mediated restraint may therefore fine-tune ILC responses under changing metabolic states.

#### 3.5.5 B cells

B cells influence obesity and insulin resistance by secreting pro-inflammatory cytokines (IL-6, TNF-α) and pathogenic antibodies. YY1 regulates B cell development, class-switch recombination, and plasma cell formation by controlling chromatin accessibility and mitochondrial metabolism (207). In contrast, RKIP restricts B cell receptor (BCR) induced activation by inhibiting Raf–MEK–ERK signaling, thereby maintaining controlled immune activation during metabolic stress.

#### 3.5.6 Microglia and tissue-resident macrophages

In the central nervous system, microglia represent specialized macrophages that orchestrate neuroinflammation associated with obesity and T2D. YY1 upregulation in microglia promotes lipid droplet accumulation, mitochondrial dysfunction, and pro-inflammatory cytokine release, thereby linking central inflammation to peripheral metabolic dysregulation (219, 232). RKIP, on the other hand, counteracts these processes by inhibiting MAPK and NF-κB pathways, preserving microglial anti-inflammatory and neuroprotective phenotypes (45, 233). Similar regulatory interactions likely occur in other tissue macrophage populations (e.g., cardiac, hepatic, and intestinal), where RKIP–YY1 crosstalk governs metabolic reprogramming and cytokine balance.

## 4 Interaction between RKIP and YY1 in immune crosstalk

### 4.1 Co-regulation of immune pathways by RKIP and YY1

The immune system relies on finely tuned signaling networks that maintain a balance between activation and suppression to ensure effective host defense while preventing excessive inflammation or autoimmunity (234). Within this regulatory architecture, RKIP and YY1 serve as key modulators, influencing immune responses through distinct yet potentially intersecting mechanisms (46).

As previously described, RKIP suppresses inflammatory signaling by inhibiting the MAPK/ERK pathway, thereby dampening immune cell activation and pro-inflammatory cytokine production (86, 235). YY1, in contrast, is a multifunctional transcription factor that can either promote or repress immune gene expression (69) depending on the cellular and promoter context. While RKIP primarily modulates upstream signaling cascades, YY1 directly governs transcriptional outputs, suggesting a framework where their combined effects may be cooperative, antagonistic, or context dependent.

Their co-regulation is particularly significant within the NF-κB signaling axis, a central pathway in the regulation of inflammation. RKIP inhibits NF-κB activation by interfering with upstream kinases responsible for phosphorylating IκBα, thereby preventing its degradation and the subsequent nuclear translocation of NF-κB subunits. In contrast, YY1 can act as either an enhancer or repressor of NF-κB activity, depending on the cellular context (116, 219) and availability of cofactors. When both RKIP and YY1 function to suppress NF-κB, their combined action may amplify anti-inflammatory effects (236). However, in settings where YY1 promotes NF-κB activity, it may antagonize RKIP’s inhibitory role, establishing a regulatory equilibrium that fine-tunes immune responses.

This balance is further modulated by the distinct yet interconnected roles of these proteins, YY1 in immune cell differentiation and gene expression, and RKIP in regulating cell survival and apoptosis (61). Collectively, RKIP and YY1 help define the immune landscape across a broad spectrum of pathological conditions, including cancer, autoimmunity, and metabolic diseases, highlighting the importance of their coordinated regulation.

#### 4.1.1 Evidence of combined effects on immune cell function and inflammation

RKIP and YY1 jointly regulate key inflammatory pathways. RKIP inhibits MAPK and NF-κB signaling in macrophages, thereby reducing cytokine production (41, 45). YY1 also suppresses inflammatory gene expression in macrophages under specific conditions (220). However, in T cells, their effects may diverge: RKIP modulates TCR signaling and can restrict effector T cell responses, while YY1 supports Treg cell function and promotes immune tolerance (203, 237). Their combined activity may help maintain immune balance by limiting excess activation while preserving Treg-mediated suppression.

In autoimmune diseases such as rheumatoid arthritis (RA) and multiple sclerosis (MS), both proteins are dysregulated (221, 238–240). Reduced RKIP expression in RA correlates with heightened NF-κB/MAPK signaling (238), whereas YY1 dysregulation impairs immune tolerance (222). Their concerted dysfunction may therefore contribute to disease exacerbation. Conversely, in cancer, tumors may exploit both proteins: YY1 can promote immune evasion by upregulating immune checkpoint molecules (e.g., PD-L1), while RKIP downregulation may enhance pro-survival pathways (223). Thus, whether RKIP and YY1 act synergistically or antagonistically depends on disease context, cell type, and environmental cues, an area that warrants further mechanistic investigation.

The precise mechanisms by which RKIP and YY1 co-regulate immune pathways remain to be fully elucidated. Key questions remain: Do they physically interact, or do they converge on common downstream targets? Are their effects cell-type-specific, or do they follow a broader regulatory pattern? Addressing these questions may uncover novel therapeutic avenues for modulating immune responses in cancer, autoimmune disorders, and inflammatory diseases. Given their broad impact on immune signaling and transcriptional regulation, RKIP and YY1 represent promising targets for precision immunotherapy aimed at restoring immune homeostasis in disease. Mechanistic Insights into Their Crosstalk.

#### 4.1.2 NF-κB and JNK axes

The NF-κB pathway serves as a central node where RKIP and YY1 exert opposing regulatory effects. RKIP inhibits NF-κB activation by targeting upstream kinases, preserving IκBα stability and preventing nuclear translocation of NF-κB components such as RelA/p65. This inhibition silences the transcription of pro-inflammatory genes (55, 71). In contrast, YY1 can act as a co-activator of NF-κB by directly interacting with RelA and recruiting CBP/p300, thereby facilitating chromatin remodeling and enhancing transcription of inflammatory genes, particularly in macrophages and microglia (232, 241).

YY1 can also promote chronic inflammation indirectly by repressing RKIP expression. This occurs through the transcriptional upregulation of Snail, which acts as a repressor of RKIP. This establishes a feed-forward inflammatory loop: NF-κB → YY1 → Snail → RKIP↓ → NF-κB↑, creating a self-perpetuating pro-inflammatory state (242).

In the JNK pathway, RKIP reduces inflammation by inhibiting upstream signaling intermediates. Conversely, YY1 may enhance JNK signaling through Smail induction, further contributing to immune dysregulation and tumor immune escape (49, 243, 244).

#### 4.1.3 Regulation of transcriptional programs and cytokines

RKIP suppresses expression of pro-inflammatory cytokines such as IFN-γ and reduces susceptibility to cytokine storms (237). In contrast, YY1 upregulates the expression of IL-6, COX-2, and TNF by directly binding to their promoters and recruiting chromatin modifiers (54). In neuroinflammatory conditions, YY1 contributes to microglial activation and has been implicated in the pathogenesis of disorders such as multiple sclerosis (MS) and Alzheimer’s disease (245, 246).

Together, RKIP and YY1 form a regulatory axis that shapes cytokine profiles and determines immune cell fate. Pharmacologic targeting of this axis, for example, by enhancing RKIP activity or inhibiting YY1, has shown promise in reversing immune resistance and promoting apoptosis in cancer models (242).

### 4.2 The RKIP-YY1 axis in immune dysregulation of metabolic disease

Metabolic diseases such as obesity, T2D, and atherosclerosis are characterized by chronic low-grade inflammation driven by persistent immune dysregulation (11). This inflammatory state arises from sustained crosstalk between immune cells and metabolic tissues, reinforcing cytokine production and promoting progressive tissue dysfunction.

Recent studies have identified RKIP and YY1 as central regulators of immune signaling in these contexts (86) (242). Their interplay coordinates both immune activation and the resolution of inflammation. Disruption of this axis has been associated with worsening inflammation, insulin resistance, and end-organ damage in metabolic disease.

In the following sections, we examine how disturbances in the RKIP–YY1 axis contribute to the pathogenesis of obesity, diabetes, and atherosclerosis, and highlight emerging opportunities for targeted therapeutic modulation.

#### 4.2.1 Regulators of immune and metabolic signaling

RKIP, a multifunctional scaffold protein, regulates key signaling cascades that govern immune responses and metabolic processes, including the MAPK, NF-κB, and GPCR pathways (39, 95). By inhibiting Raf-1-mediated activation of the MAPK cascade, RKIP suppresses pro-inflammatory and proliferative responses (30). It also binds to NIK, thereby dampening NF-κB activity and reducing the production of cytokines such as TNF and IL-6 (45, 71).

In contrast, the transcription factor YY1 is broadly expressed and exerts dual regulatory roles, either activating or repressing immune and metabolic genes (69) depending on cellular context. For instance, YY1 enhances NF-κB-driven cytokine expression while suppressing IL-10 signaling during metabolic stress (116, 219). The regulatory axis formed by RKIP and YY1 is thus critical for maintaining immune-metabolic homeostasis. Under metabolic stress, decreased RKIP expression leads to unchecked YY1-mediated inflammation, exacerbating disease progression.

#### 4.2.2 Obesity and adipose tissue inflammation

Obesity promotes adipose tissue expansion, which is accompanied by hypoxia, ER stress, and the infiltration and accumulation of proinflammatory M1 macrophages (247). In lean adipose tissue, RKIP plays a protective role by inhibiting NF-κB and MAPK signaling pathways (87). It stabilizes the interaction between IκB and NF-κB, thereby preventing NF-κB nuclear translocation and downstream inflammatory gene expression (92, 116). However, obesity-associated oxidative stress and hyperinsulinemia downregulate RKIP expression, weakening its regulatory control and allowing unchecked YY1 activity (248).

In adipocytes and adipose tissue macrophages (ATMs), elevated YY1 activity upregulates the expression of pro-inflammatory genes such as TNF, IL-6, and MCP-1 (54). Simultaneously, YY1 represses PPARγ activity, thereby inhibiting adipocyte differentiation and promoting a shift away from M2 macrophage polarization (217, 249, 250). This inflammatory environment enhances NLRP3 inflammasome activation, perpetuating chronic inflammation and contributing to the development of insulin resistance (251).

#### 4.2.3 Pancreatic β-cell dysfunction

In T2D, inflammatory cytokines disrupt insulin secretion and induce β-cell apoptosis (252, 253). RKIP may influence apoptosis through ASK1-mediated pathways, although this mechanism remains underexplored. Conversely, YY1 supports mitochondrial integrity and ATP production in β-cells, thereby improving insulin secretion and delaying disease onset (254).

#### 4.2.4 Vascular inflammation and atherosclerosis

In vascular tissues, RKIP regulates smooth muscle cell migration and suppresses inflammatory signaling (255). YY1 contributes to foam cell formation by upregulating PCSK9 and modulating LDLR pathways, facilitating oxLDL uptake in macrophages (33).

## 5 Tissue-contexts for RKIP and YY1 interactions in inflammation and metabolic disease

The pro-inflammatory cytokines regulated by the RKIP-YY1 axis are key mediators of metabolic inflammation, contributing to insulin resistance and tissue dysfunction in obesity, T2D, and their associated complications and comorbidities (11, 256). These cytokines are primarily secreted by macrophages and other innate immune cells in response to nutrient excess and lipotoxicity.

Across tissue compartments, RKIP modulates immune responses by inhibiting key inflammatory signaling pathways such as MAPK and NF-κB. RKIP deficiency has been associated with enhanced production of pro-inflammatory cytokines, including IL-6 and IL-1β, thereby exacerbating inflammatory responses (183). YY1, by contrast, can function as a co-activator of NF-κB, augmenting transcription of pro-inflammatory genes. Overexpression of YY1 has been linked to increased inflammation in multiple tissues, promoting metabolic dysregulation (257). While these regulatory mechanisms are active across various tissues, they carry specific implications in metabolically active organs, such as adipose tissue, liver, and skeletal muscle, under conditions of dysmetabolism characteristic of obesity and T2D.

In adipose tissue, the influx of pro-inflammatory macrophages during obesity is likely modulated by the RKIP-YY1 axis. Notably, RKIP plays a crucial role in regulating macrophage polarization, with its deficiency associated with heightened inflammatory responses (114). Conversely, YY1 influences adipocyte differentiation and promotes pro-inflammatory gene expression when dysregulated (217).

Chronic inflammation in the liver contributes to the progression of MASLD to steatohepatitis. Although RKIP’s role has not been directly demonstrated in MASLD models, studies in other liver injury contexts indicate that RKIP-mediated suppression of MAPK and NF-kB pathways exerts protective effects (258, 259). YY1 is implicated in hepatic lipid metabolism and inflammatory signaling, thereby influencing the development and progression of liver disease (260).

Skeletal muscle plays a central role in regulating glucose homeostasis due to its high insulin sensitivity and rapid glucose uptake from the circulation. Inflammatory cytokines impair insulin signaling in muscle tissue, contributing to insulin resistance. RKIP modulates inflammatory signaling pathways and may help attenuate muscle inflammation (261). Meanwhile, YY1 regulates genes essential for mitochondrial function and energy metabolism, and its dysregulation in skeletal muscle is associated with mitochondrial dysfunction and broader metabolic disturbances (262).

### 5.1 RKIP and YY1 in immune cell reprogramming during metabolic disease remission

Recent research emphasizes that remission of metabolic diseases such as obesity, type 2 diabetes (T2D), and cardiovascular disease (CVD) is not merely a metabolic adjustment but involves profound immune remodeling. Immune cells that propagate chronic inflammation during disease progression undergo reprogramming under effective therapeutic or lifestyle interventions, shifting toward anti-inflammatory or reparative states. RKIP and YY1, both key regulators of immune and metabolic transcriptional programs, appear central in orchestrating this process.

#### 5.1.1 Obesity and weight-loss induced immune remodeling

In obesity, adipose tissue is enriched in proinflammatory macrophages (M1-like), CD8^+^ T cells, and Th1 lymphocytes that contribute to insulin resistance and local inflammation (263, 264). Weight loss, whether achieved through caloric restriction, bariatric surgery, or thermogenic agents, restores immune balance by promoting anti-inflammatory M2-like macrophages, regulatory T cells (Tregs), and eosinophils (263, 265). RKIP expression rises in adipose macrophages and stromal cells following metabolic improvement, coinciding with suppressed NF-κB and JNK activation and improved insulin sensitivity (71). By inhibiting MAPK/NF-κB pathways, RKIP enhances oxidative phosphorylation and lipid clearance genes. Conversely, YY1, which is elevated in obese adipose tissue, is downregulated with weight loss, releasing repression on PGC-1α and mitochondrial biogenesis genes (77, 266). Collectively, enhanced RKIP and reduced YY1 activity promote transcriptional reprogramming toward a metabolically favorable, anti-inflammatory adipose immune environment.

#### 5.1.2 Type 2 diabetes and pharmacological reprogramming of tissue immunity

In T2D, immune infiltration of pancreatic islets, skeletal muscle, and even the hypothalamus contributes to insulin resistance and β-cell failure (267). Antidiabetic therapies such as metformin, GLP-1 receptor agonists, and SGLT2 inhibitors restore immune and metabolic homeostasis (268). Metformin activates AMPK and indirectly increases RKIP activity by suppressing NF-κB and STAT3 signaling, leading to decreased macrophage and T-cell inflammatory cytokine production (269). In contrast, hyperglycemia-driven YY1 upregulation in diabetic tissues represses oxidative and mitochondrial genes; normalization of glucose levels reverses this effect. In pancreatic β-cells, reduced YY1 expression after glycemic control correlates with enhanced insulin gene transcription and decreased ER stress (270). Increased RKIP expression in β-cells and immune cells may further limit apoptosis through inhibition of MAPK cascades (176). Thus, therapeutic rebalancing of RKIP–YY1 signaling contributes to immune normalization and improved insulin action in T2D remission.

#### 5.1.3 Cardiovascular disease and immune cell plasticity under therapeutic intervention

In atherosclerosis, macrophage-derived foam cells and activated vascular immune cells perpetuate plaque inflammation and instability (271, 272). Clinical interventions such as statins, β-blockers, and ACE inhibitors exert anti-inflammatory effects beyond their metabolic or hemodynamic actions (273, 274). Statins indirectly enhance RKIP levels in endothelial and monocyte-derived cells, strengthening inhibition of ERK/NF-κB signaling and promoting cholesterol efflux via ABCA1/ABCG1 (275). ACE inhibitors similarly upregulate RKIP, contributing to decreased vascular inflammation. Conversely, YY1 downregulation by statins alleviates repression of ABCA1 and LXRα transcription, favoring lipid efflux and plaque regression. β-blockers, by attenuating catecholamine-induced YY1 activation, indirectly support RKIP-mediated suppression of inflammatory signaling in foam cells. These findings indicate that modulation of the RKIP–YY1 axis contributes to immune and metabolic restoration within the vascular microenvironment.

## 6 Therapeutic implications and future directions

Targeting the RKIP-YY1 axis holds promise for mitigating metabolic inflammation and may offer effective treatment strategies for inflammatory disorders. Pharmaceutical RKIP enhancers, YY1 inhibitors, and peptides designed to disrupt RKIP-YY1 interactions could help restore immune homeostasis. However, because YY1 regulates numerous essential cellular functions, achieving tissue-specific delivery will be critical to minimize off-target effects. While no direct RKIP-targeting compounds have been developed to date, several direct YY1 inhibitors have shown efficacy in preclinical studies (276). Non-pharmacological interventions such as exercise and caloric restriction have also been shown to upregulate RKIP expression, supporting their use as adjunctive strategies in managing metabolic inflammation (277, 278).

### 6.1 Targeting RKIP and YY1 for metabolic disease therapy

Modulating the activity of RKIP and YY1 represents a promising therapeutic strategy for the treatment of metabolic diseases. Enhancing RKIP expression or function may attenuate inflammatory signaling pathways and improve insulin sensitivity (45). Conversely, inhibiting YY1 activity could suppress the transcription of pro-inflammatory genes, thereby mitigating the chronic inflammation characteristic of metabolic disorders. YY1’s involvement in cellular metabolism (217, 254), including mitochondrial function and adipogenesis, further underscores its potential as a therapeutic target.

Although specific pharmacological agents targeting RKIP are still under investigation, therapeutic strategies may include gene therapy or small molecules designed to enhance RKIP expression or stabilize the protein. For YY1, potential interventions may involve inhibitors that disrupt their interactions with transcriptional co-activators or its DNA-binding activity.

Currently, the development of RKIP and YY1 targeted therapies is primarily focused on oncology. Nonetheless, given their functional relevance across key metabolic tissues and their regulatory roles throughout the spectrum of metabolic disease, both RKIP and YY1 represent viable and compelling targets for therapeutic intervention in obesity and T2D.

### 6.2 Immune modulation as a therapeutic strategy

Targeting immune pathways continues to offer significant promise in the treatment of metabolic diseases. For example, therapies that neutralize pro-inflammatory cytokines, such as IL-1β antagonists, have shown efficacy in improving glycemic control among patients with T2D (279, 280). Additionally, agents that modulate immune cell recruitment and activation within metabolic tissues may help resolve chronic inflammation and restore metabolic homeostasis.

Nevertheless, challenges remain, particularly with regard to achieving tissue-specific modulation while minimizing systemic immunosuppression and associated adverse effects. Further research is required to develop immune interventions that fine-tune inflammatory responses without impairing host defense. Emerging technologies, such as bispecific antibodies for targeted delivery (281) and antisense oligonucleotides (ASO) for precise transcriptional interference (282), are expanding these possibilities. While ASOs have been designed and tested against relevant targets such as PPARG (283), they have not yet been developed to specifically modulate RKIP or YY1, representing a potential area for future therapeutic innovation.

### 6.3 Future research directions

Given the ubiquitous expression of RKIP and YY1, and their pivotal roles in immune and metabolic regulation, several key areas merit further investigation. First, deeper elucidation is needed regarding the molecular mechanisms through which RKIP and YY1 regulate immune responses within specific tissue contexts, particularly those critical to systemic metabolic homeostasis, across different stages of disease progression.

Second, the development of selective modulators of RKIP and YY1 activity remains an important goal. Emerging scalable technologies, such as monoclonal or bispecific antibodies, decoy peptides, or ASOs, offer promising platforms. However, their therapeutic efficacy, pharmacokinetics, and safety profiles must be carefully evaluated within metabolic disease models.

Third, a better understanding of the crosstalk between immune cells and metabolic pathways across tissues will help identify novel downstream targets of RKIP or YY1. For instance, previously discussed targets like PPARγ could serve as actionable nodes for therapeutic modulation.

Finally, future research should investigate the utility of RKIP and YY1 as biomarkers for disease progression and treatment response. Their potential roles across a broader range of metabolic conditions, beyond obesity and T2D, may further inform precision medicine approaches and support translational implication.

## 7 Conclusion

The pathogenesis of metabolic diseases is now widely recognized as an immunometabolic disorder, wherein chronic low-grade inflammation, driven by dysregulated immune signaling, directly contributes to tissue dysfunction and systemic insulin resistance. Within this framework, RKIP and YY1 emerge as two pivotal regulators that coordinate immune cell activation, inflammatory mediator production, and tissue responses to metabolic stress.

RKIP functions primarily as a negative regulator of pro-inflammatory signaling pathways, including MAPK, NF-κB, and GPCR cascades. By inhibiting RAF and NF-κBn inducing kinases, RKIP restricts the propagation of cytokine and chemokine expression, thereby preserving tissue homeostasis and limiting immune overactivation. In contrast, YY1 acts as a transcriptional integrator of inflammatory and metabolic signals. Its dual capacity to either activate or repress gene expression enables context-dependent control over immune cell differentiation, chromatin architecture, and cytokine responses under conditions of nutrient excess and cellular stress. The reciprocal regulation between RKIP and YY1, mediated through feedback loops involving NF-κB and Snail, adds an additional layer of control to inflammatory signaling. This RKIP–YY1 axis operates across key metabolic tissues, including adipose tissue, liver, skeletal muscle, and pancreas, to regulate macrophage polarization, cytokine production, adipogenesis, mitochondrial function, and β-cell survival. Disruption of this balance, as observed in obesity and T2D, favors YY1-mediated inflammation and metabolic dysfunction, while RKIP downregulation eliminates critical inhibitory checkpoints.

Therapeutically, targeting the RKIP–YY1 axis represents a promising avenue for intervention. Enhancing RKIP function or stability could suppress chronic inflammation and restore insulin sensitivity, while YY1 inhibition may attenuate the transcription of genes that drive inflammatory and metabolic dysfunction. Early-stage studies point to the potential of small molecules, gene therapies, ASOs, and decoy peptides to modulate this axis. Nonetheless, the ubiquitous expression and pleiotropic roles of both proteins underscore the need for tissue-specific or inducible delivery strategies to minimize off target effects.

Future research should focus on the mechanistic dissection of RKIP and YY1 functions in defined immune cell subsets and metabolically active tissues. High-resolution multi-omics approaches, such as single-cell transcriptomics, spatial proteomics, and integrative network modeling, will be critical to mapping their dynamic roles throughout disease progression. Additionally, extending the study of RKIP–YY1 axis to other metabolic contexts, including MASLD, diabetes complications, and cardiometabolic diseases, will help clarify its translational significance.

In conclusion, RKIP and YY1 function as central nodes at the intersection of immunity and metabolism. Their reciprocal regulation and shared governance over inflammatory signaling define a powerful immunometabolic regulatory axis. Understanding this axis may offer transformative therapeutic opportunities for the treatment of chronic metabolic disorders and their complications.

## Glossary

3T3-L1: Mouse Preadipocyte Cell Line
ABCA1/ABCG1: ATP-Binding Cassette Transporters A1 and G1
ACE: Angiotensin-Converting Enzyme
Act1: NF-κB Activator 1 (also called TRAF3IP2)
ASC: Apoptosis-Associated Speck-Like Protein Containing a CARD
ASK1: Apoptosis Signal-Regulating Kinase 1
ATP: Adenosine Triphosphate
BAFFR: B-Cell Activating Factor Receptor
BCR: B-Cell Receptor
C/EBPβ: CCAAT/Enhancer-Binding Protein Beta
C₂H₂: Cysteine₂–Histidine₂
CBP: CREB-Binding Protein
CD40: Cluster of Differentiation 40
CHREBP: Carbohydrate-Responsive Element-Binding Protein
COX-2: Cyclooxygenase-2
CpG: Cytosine-Phosphate-Guanine Motif
CVD: Cardiovascular Disease
DCs: Dendritic Cells
DNA: Deoxyribonucleic Acid
EMT: Epithelial-to-Mesenchymal Transition
ER: Endoplasmic Reticulum
ERK: Extracellular Signal–Regulated Kinase
FASN: Fatty Acid Synthase
GK-rich: Glycine–Lysine–Rich
GLP-1: Glucagon-Like Peptide-1
GPCR: G Protein Coupled Receptor
GRK2: G Protein Coupled Receptor Kinase 2
HDACs: Histone Deacetylases
HK2: Hexokinase 2
IFN-β: Interferon Beta
IFN-γ: Interferon-gamma
IKK: IκB Kinase
IL-10: Interleukin-10
IL-17R: Interleukin-17 Receptor
IL-1R: Interleukin-1 Receptor
IL-1β: Interleukin-1 beta
IL-6: Interleukin-6
IRS: Insulin Receptor Substrate
JNK: c-Jun N-terminal Kinase
KO: Knockout
LDLR: Low-Density Lipoprotein Receptor
LPS: Lipopolysaccharide
LTβR: Lymphotoxin-β
LXRα: Liver X Receptor Alpha
M1: M1-like Macrophages
M2: M2-like Macrophages
MAPK: Mitogen-Activated Protein Kinase
MASLD: Metabolic Dysfunction-Associated Steatotic Liver Disease
MCP-1: Monocyte Chemoattractant Protein-1
MEK: MAPK/ERK Kinase
MS: Multiple Sclerosis
mTOR: Mechanistic Target of Rapamycin
mTORC1: mTOR Complex 1
MyD88: Myeloid Differentiation Primary Response 88
NFAT: Nuclear Factor of Activated T Cells
NF-κB: Nuclear Factor kappa-light-chain-enhancer of Activated B Cells
NIK: NF-κB-Inducing Kinase
NLRP3: NOD-Like Receptor Protein 3
oxLDL: Oxidized Low-Density Lipoprotein
PAMPs: Pathogen-Associated Molecular Patterns
PCSK9: Proprotein Convertase Subtilisin/Kexin Type 9
PD-L1: Programmed Death-Ligand 1
PEBP1: Phosphatidylethanolamine-Binding Protein 1
PFKFB3: 6-Phosphofructo-2-Kinase/Fructose-2,6-Bisphosphatase 3
PGC-1α: Peroxisome Proliferator-Activated Receptor Gamma Coactivator-1 Alpha
PKC: Protein Kinase C
PPARγ: Peroxisome Proliferator-Activated Receptor Gamma
PRC1/2: Polycomb Repressive Complex 1 and 2
RA: Rheumatoid Arthritis
RAF: Rapidly Accelerated Fibrosarcoma Kinase
RelB: NF-κB Family Member RelB
REPO[HA2.1]: REPO Motif (Histone Association Motif 2.1)
RKIP: Raf Kinase Inhibitor Protein
RNA: Ribonucleic Acid
SCD1: Stearoyl-CoA Desaturase 1
Ser153: Serine Residue 153
SGLT2: Sodium–Glucose Cotransporter 2
SLE: Systemic Lupus Erythematosus
Snail: Zinc-Finger Transcriptional Repressor SNAI1
Src[HA3.1]: Src Family Kinases (Histone Association Region 3.1)
SREBP1: Sterol Regulatory Element-Binding Protein 1
STAT3: Signal Transducer and Activator of Transcription 3
T1D: Type 1 Diabetes
T2D: Type 2 Diabetes
TAK1: Transforming Growth Factor Beta–Activated Kinase 1
TBK1: TANK-Binding Kinase 1
TCR: T-Cell Receptor
Th1: T Helper Type 1 Cells
TLR: Toll-Like Receptor
TNF: Tumor Necrosis Factor
TNFR: Tumor Necrosis Factor Receptor
TRAF6: TNF Receptor–Associated Factor 6
Treg: Regulatory T Cell
YY1: Yin Yang 1
β-blocker: Beta-Adrenergic Receptor Antagonist
